# Relative abundance of the *Prevotella* genus within the human gut microbiota of elderly volunteers determines the inter-individual responses to dietary supplementation with wheat bran arabinoxylan-oligosaccharides

**DOI:** 10.1186/s12866-020-01968-4

**Published:** 2020-09-14

**Authors:** Wing Sun Faith Chung, Alan W. Walker, Douwina Bosscher, Vicenta Garcia-Campayo, Josef Wagner, Julian Parkhill, Sylvia H. Duncan, Harry J. Flint

**Affiliations:** 1 Gut Health Group, Rowett Institute, University of Aberdeen, Foresterhill, Aberdeen, Scotland AB25 2ZD UK; 2 Cargill R&D Centre Europe BVBA, Havenstraat 84, B-1800 Vilvoorde, Belgium; 3 Cargill, Incorporated, PO Box 9300, Minneapolis, MN 55440-9300 USA; 4 Pathogen Genomics Group, Wellcome Sanger Institute, Hinxton, Cambridgeshire, CB10 1SA UK; 5 Department of Veterinary Medicine, University of Cambridge, Madingley Road, Cambridge, CB3 0ES UK

**Keywords:** Gut microbiota, Diversity, *Bacteroides*, *Prevotella*, Bifidobacteria, Wheatbran extract, Arabinoxylan oligosaccharides (AXOS), Short chain fatty acids, Propionate, Butyrate

## Abstract

**Background:**

The human colon is colonised by a dense microbial community whose species composition and metabolism are linked to health and disease. The main energy sources for colonic bacteria are dietary polysaccharides and oligosaccharides. These play a major role in modulating gut microbial composition and metabolism, which in turn can impact on health outcomes.

**Results:**

We investigated the influence of wheat bran arabinoxylan oligosaccharides (AXOS) and maltodextrin supplements in modulating the composition of the colonic microbiota and metabolites in healthy adults over the age of 60. Male and female volunteers, (*n* = 21, mean BMI 25.2 ± 0.7 kg/m^2^) participated in the double-blind, cross over supplement study. Faecal samples were collected for analysis of microbiota, short chain fatty acids levels and calprotectin. Blood samples were collected to measure glucose, cholesterol and triglycerides levels. There was no change in these markers nor in calprotectin levels in response to the supplements. Both supplements were well-tolerated by the volunteers. Microbiota analysis across the whole volunteer cohort revealed a significant increase in the proportional abundance of faecal *Bifidobacterium* species (*P* ≤ 0.01) in response to AXOS, but not maltodextrin, supplementation. There was considerable inter-individual variation in the other bacterial taxa that responded, with a clear stratification of volunteers as either *Prevotella*-plus (*n* = 8; > 0.1% proportional abundance) or *Prevotella*-minus (*n* = 13; ≤0.1% proportional abundance) subjects founded on baseline sample profiles. There was a significant increase in the proportional abundance of both faecal *Bifidobacterium* (*P* ≤ 0.01) and *Prevotella* species (*P* ≤ 0.01) in *Prevotella*-plus volunteers during AXOS supplementation, while *Prevotella* and *Bacteroides* relative abundances showed an inverse relationship. Proportional abundance of 26 OTUs, including bifidobacteria and *Anaerostipes hadrus,* differed significantly between baseline samples of *Prevotella*-plus compared to *Prevotella*-minus individuals.

**Conclusions:**

The wheat bran AXOS supplementation was bifidogenic and resulted in changes in human gut microbiota composition that depended on the initial microbiota profile, specifically the presence or absence of *Prevotella* spp. as a major component of the microbiota. Our data therefore suggest that initial profiling of individuals through gut microbiota analysis should be considered important when contemplating nutritional interventions that rely on prebiotics.

**Trial registration:**

Clinical trial registration number: NCT02693782. Registered 29 February 2016 - Retrospectively registered, https://clinicaltrials.gov/ct2/show/NCT02693782?term=NCT02693782&rank=1

## Background

The human large intestine is colonised by a dense microbial community, comprised of a large number of different species mainly belonging to the Bacteroidetes, Firmicutes and Actinobacteria phyla [1], and microbial composition can have a major impact on health outcomes [2]. The composition of the gut microbiota is considered to be driven primarily by diet, geographical location and age [3, 4]. Actinobacteria typically account for around 5% of the microbiota in faecal samples from healthy adults and *Bifidobacterium* is one of the most abundant constituent genera in this phylum [5]. There has been particular interest in bifidobacteria because of their use as probiotics and as targets for prebiotics [6]. The abundance of bifidobacteria is diminished in the elderly [6, 7] and low abundance of bifidobacteria is also associated with health disorders including inflammatory bowel disease [8].

The species composition of the colonic microbiota is also likely to be driven by dietary macronutrients, in particular non-digestible carbohydrates derived from plant fibre [9, 10]. Among the Bacteroidetes phylum, the two most prevalent genera in the human colon are generally *Bacteroides* and *Prevotella.* It has been proposed that high dietary fibre intake is associated with a *Prevotella* dominated microbiota [11], whereas high intake of fat and protein is associated with a *Bacteroides* dominated microbiota, based on a survey of 98 US adults [11–13]. Indeed, microbiota profiling studies carried out in agrarian, and high-fibre consuming, populations around the World have consistently reported that the *Prevotella* genus is more dominant than in urbanised societies typically consuming lower fibre diets, where *Bacteroides* typically appear to be more predominant [11, 14].

Human studies allow us to gain unique information on the dynamics of the gut microbial community and have revealed that changes in diet-responsive species can occur rapidly, often within a few days of the dietary interventions, with concomitant alterations in microbial metabolite formation [15–18]. There is a pressing need to identify the types of bacterial taxa that can be promoted by specific dietary components, and in turn how dietary-induced shifts in microbiota composition and activities might impact on the host [2, 4, 19, 20].

It has been demonstrated that arabinoxylan oligosaccharides (AXOS) have beneficial properties across different age groups including promotion of faecal bifidobacteria abundance and overall short chain fatty acid levels [21–26]. AXOS are short-oligosaccharides obtained from the hydrolysis of arabinoxylans that are composed of a linear backbone of β-1,4-linked D-xylopyranosyl residues (xylose), some of which are substituted with α-L-arabinofuranosyl residues (arabinose) that can be ester-linked with ferulic acid [27].

Here we investigated the impact of dietary supplementation with wheat bran-derived AXOS or maltodextrin on gut microbiota composition and metabolites in 21 free living, elderly volunteers. AXOS, but not maltodextrin, supplementation generally increased bifidobacteria proportional abundance, but faecal microbiota responses to AXOS differed significantly between individuals who were *Prevotella*-positive (*Prevotella*-plus group) and those who were *Prevotella*-negative (*Prevotella*-minus group) at baseline. The eight volunteers presenting as *Prevotella*- plus showed a significant increase in faecal total short chain fatty acids and propionate during the AXOS supplementation. These findings have important consequences for our understanding of inter-individual responses to a wheat bran AXOS dietary supplement and are likely to have important consequences for health.

## Results

### Demographics and metadata for human volunteers

**Fig. 1:**
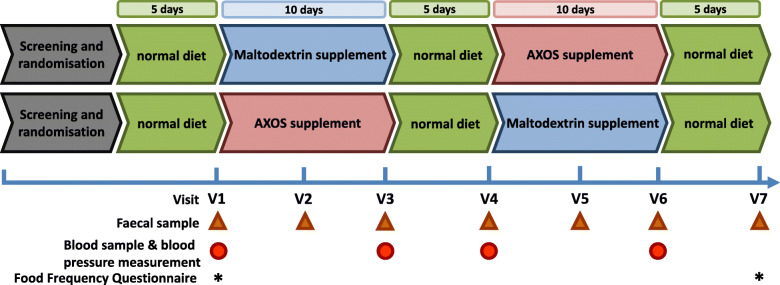
Schematic of the cross-over, randomised, double-blinded design of the human dietary supplementation study, incorporating the addition of Wheat Bran Extract arabinoxylan oligosaccharide (AXOS) and maltodextrin. Control periods, when volunteers were not receiving supplements (5 days each) were included before, in between, and after the supplementation periods (10 days per supplement). Study visits and sample collection days are indicated. Faecal samples were collected at each visit. Blood samples and blood pressure measurements were collected at the start and end of each supplementation period. Food frequency questionnaires were completed at the beginning and end of the study

Free living male and female (BMI 20–32 kg/m^2^), elderly volunteers that met the inclusion criteria for the study (see Materials and Methods) were given either AXOS extracted from wheat bran or maltodextrin supplements in a randomised, cross-over, double-blinded manner. Maltodextrin was chosen as a comparator and the two supplementation periods had a duration of ten days each and were separated by washout periods of 5 days each. Faecal samples were collected every 5 ± 1 days. Blood collection and blood pressure measurements were performed at the beginning and the end of each supplementation period (Fig. 1).

Supplements of both wheat bran AXOS (Brana Vita® 21,760) and maltodextrin were provided in sachets containing 5 g of the powdered supplement and consumed three times per day to give to total of 15 g/d (97% dry matter). The two supplements were delivered in a double-blinded manner. Compliance was determined by weigh back of residual powders in the used sachets. The compliance for the supplements in the AXOS and maltodextrin periods were 96 and 95.9% respectively.

Male and female volunteers aged 60 years and above, with a body mass index (BMI) between 20 and 32 kg/m^2^ (which is within the normal to slightly obese BMI range) were recruited locally (Aberdeen, UK). Of the 26 volunteers who attended for screening five volunteers did not participate further in the study due to unspecified personal reasons (volunteers 007, 012, 015, 016, and 017). Twenty-one volunteers (average age of 69.7 years and an average BMI of 25.2 kg/m^2^) completed the study (eight males and thirteen females) (Table 1). The volunteers were randomised into two groups. Group 1 (*n* = 8) received the maltodextrin supplement in the first period then AXOS supplement in the second period, whereas group 2 (*n* = 13) received the AXOS supplement in the first period then the maltodextrin supplement in the second period. All twenty-one volunteers who completed the study provided a complete set of seven faecal samples, except for volunteer 008 (Additional file 1: Table S1) who provided five faecal samples (samples lacking for visits 1 and 5). All volunteers, with the exception of volunteer 022, provided blood samples.

**Table 1:** Summary of the completed volunteer demographics (*n* = 21), showing gender, age, height, weight and BMI. Values are shown as mean ± SEM and range

	MEAN ± SEM (Range)			
	Age (yrs)	Height (m)	Weight (kg)	BMI (kg/m^**2**^)
Males (*n* = 8)	68.3 ± 1.74 (61–75)	1.7 ± 0.02 (1.608–1.773)	75.63 ± 3.76 (57.35–87.85)	26.14 ± 1.01 (22.18–30.18)
Females (n = 13)	67.31 ± 1.26 (60–74)	1.61 ± 0.01 (1.493–1.693)	63.61 ± 2.66 (48.9–81.9)	24.6 ± 0.95 (18.98–31.25)
All (n = 21)	67.67 ± 1 (60–75)	1.64 ± 0.02 (1.493–1.773)	68.19 ± 2.49 (48.9–87.85)	25.18 ± 0.71 (18.98–31.25)

**Fig. 2:**
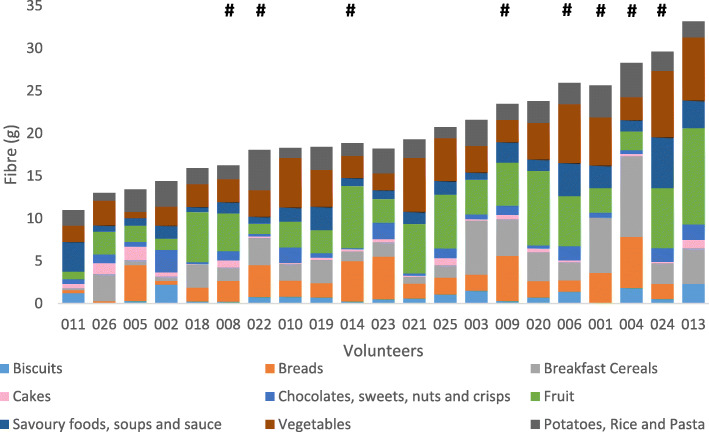
Habitual dietary fibre intake from different dietary sources per volunteer from data collected via food frequency questionnaires. The total intake of dietary fibre ranged from 10.6 to 38.5 g/day with an average of 20.9 g/day across the volunteers. Currently, the recommended average fibre intake for adults in the UK is 30 g/day. (https://www.gov.uk/government/publications/sacn-carbohydrates-and-health-report). # Indicates *Prevotella-*plus individuals

Habitual dietary intakes were recorded twice (beginning and end) within the study period using food frequency questionnaires (Fig. 1). The mean macronutrient content of the volunteers’ daily diet comprised 61.8% carbohydrate (which included sugars), 17.9% protein and 17.3% fat (in addition to 3% minerals and vitamins) (*n* = 42 representing two measurements per volunteer). Habitual dietary fibre intake per volunteer was variable and ranged from 11 to 39 g/day, with an average of 21 g/day across all volunteers (Fig. 2).

### Gastrointestinal tolerance

Gastrointestinal tolerance was recorded daily by the volunteers (*n* = 21) throughout the study and the symptoms (as defined in the Materials and Methods) were scored between 0 and 3 for no more, slightly more, noticeably more, or considerably more symptoms than usual. There were no reported ill-effects for the two treatment periods and all score changes were less than 1, indicating very mild to no effect, with excellent compliance for the supplements in the AXOS and maltodextrin periods (data not shown). Stool appearance was also recorded daily and the majority of the samples from all three periods (washout control period, AXOS supplementation and maltodextrin supplementation) were reported as normal.

### Effect of arabinoxylan-oligosaccharide (AXOS) on the human gut microbial communities

The intestinal bacterial communities were analysed using Illumina MiSeq sequencing of PCR amplified (V1-V2 region only) 16S rRNA genes from faecal samples, including samples from the initial washout, intermediate and end time points from the first supplement period, the middle washout, intermediate and end time points from the second period and the final washout. A total of 152 samples were analysed overall, resulting in a total of 573,488 sequences (Additional file 2: Table S2).

**Fig. 3:**
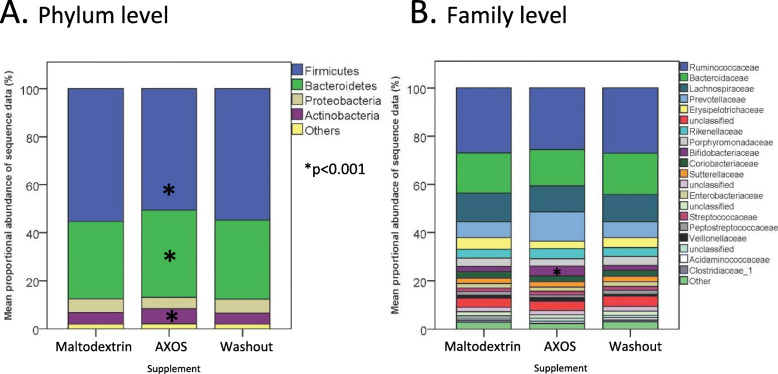
Mean proportional abundance of bacteria at (**a**) the phylum and (**b**) the family level during the maltodextrin, AXOS and control (washout) periods determined by Illumina sequencing. (* = For panel A, *p* < 0.001; for panel B, *p* < 0.05)

As is typically observed with human gut microbiota studies, there was a large degree of inter-individual variation in response to dietary supplementation, and the most abundant phyla were Firmicutes, Bacteroidetes, Actinobacteria and Proteobacteria [4, 28]. However, across the entire cohort of volunteers there was an increase in proportional abundance of Actinobacteria during the AXOS supplementation period compared to both the maltodextrin supplementation (Metastats, *p* < 0.001) and washout control periods (*p* < 0.001). The mean proportional abundance of Bacteroidetes was also significantly higher during AXOS supplementation (*p* < 0.001) with a concomitant proportional decrease in Firmicutes (*p* < 0.001; Fig. 3a).

This observed increase in the proportional abundance of Actinobacteria was mainly driven by an increase at the family level of Bifidobacteriaceae when compared to the washout control and maltodextrin supplementation periods (*p* < 0.05; Fig. 3b).

### Stratification of volunteers based on faecal microbiota composition

Further inspection of the microbiota data indicated a distinct split in the types of bacteria that responded to the AXOS supplementation, which depended fundamentally on the baseline microbiota composition of the volunteers. These data revealed a subgroup of eight volunteers who were *Prevotella*-positive (plus) (volunteers 1, 4, 6, 8, 9, 14, 22 and 24), as their baseline samples showed > 0.1% proportional abundance of total Prevotellaceae (range 2.7–28.4%) whilst the remaining 13 volunteers (2, 3, 5, 10, 11, 13, 18, 19, 20, 21, 23, 25 and 26) had low (cut-off set at ≤0.1%) or no detectable *Prevotella* spp. at their baseline sample. The proportional abundance of *Bacteroides* spp. was significantly higher in the *Prevotella*-minus than in the *Prevotella*- plus group (OTU0003 *Bacteroides uniformis p* = 0.001 and OTU0020 *Bacteroides cellulosilyticus p* = 0.001) (Additional file 3: Table S3). Overall, 26 of the most abundant operational taxonomic units (OTUs) (those comprising > 0.5% of sequences in baseline samples) differed significantly between the *Prevotella*-plus and *Prevotella*-minus groups (Additional file 3: Table S3).

**Fig. 4:**
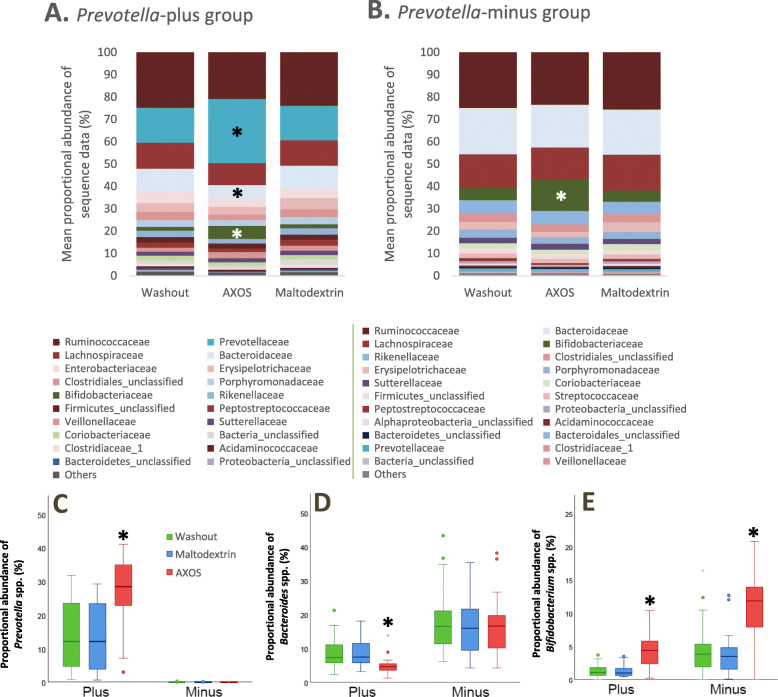
Mean proportional bacterial abundance changes at (**a**) the family level for the *Prevotella*-plus group (*Prevotellaceae p* = 0.002, Bifidobacteriaceae *p* = 0.0002, Bacteroidaceae *p* = 0.004) and (**b**) *Prevotella*-minus group (Bifidobacteriaceae *p* < 0.0001) during maltodextrin, AXOS supplementation and washout periods. Mean proportional abundance of (**c**) *Prevotella* spp. (*Prevotella*-plus group genus level, increased during AXOS supplementation *p* = 0.002), (**d**) *Bacteroides* spp. (*Prevotella*-plus group genus level, decreased during AXOS supplementation *p* = 0.004), and (**e**) *Bifidobacterium* spp. (*Prevotella*-plus group genus level, increased during AXOS supplementation *p* = 0.0001, *Prevotella*-minus group, AXOS *p* < 0.0001) during the maltodextrin, AXOS and washout periods. *p ≤ 0.01, significance from LEfSe analysis comparing across all three supplement periods

**Fig. 5:**
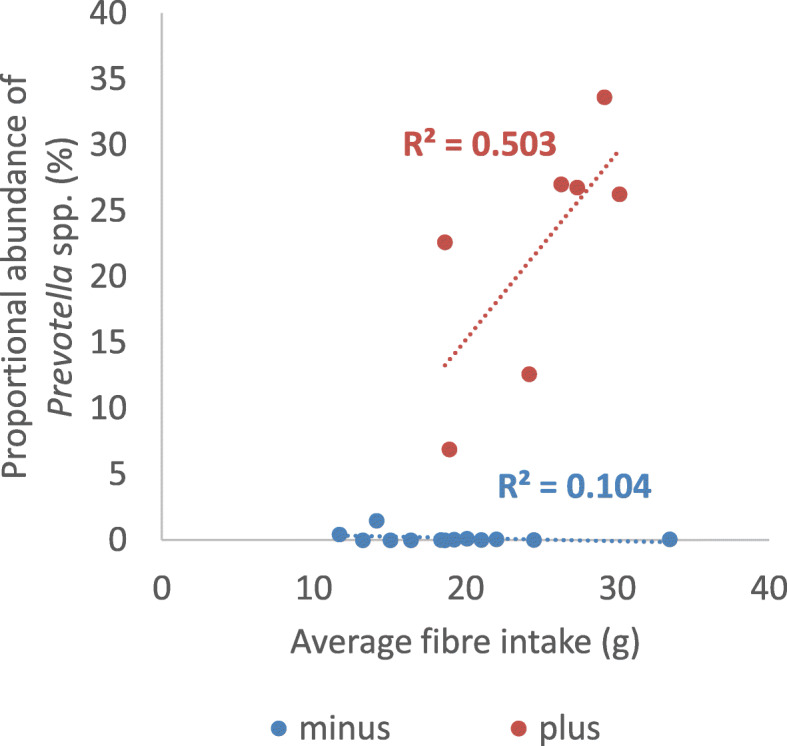
Correlation of habitual fibre intake and *Prevotella* proportional abundance from first washout sample (baseline) in the *Prevotella*-plus group of volunteers (R^2^ = 0.503, *p* = 0.07). One volunteer in the *Prevotella*-plus group (volunteer 8) did not provide a first washout sample and therefore was not included in this figure

Analysis of the microbiota in the *Prevotella*-plus group at the family level showed a significant increase in Prevotellaceae proportional abundance during the AXOS supplementation period compared to both the washout and maltodextrin periods (LEfSe, *p* = 0.002, validated with Wilcoxon-ranked test *p* = 0.014) (Additional file 4: Table S4) and a decrease in Bacteroidaceae proportional abundance (LEfSe, *p* = 0.005, not significant with Wilcoxon-ranked test) (Fig. 4a-d) compared to baseline. The proportional abundance of both the Bifidobacteriaceae family and *Bifidobacterium* genus were significantly higher during the AXOS period compared to the maltodextrin and control (baseline) periods for both the *Prevotella*-plus and *Prevotella*-minus group of volunteers (LEfSe, *p* < 0.0001) (Fig. 4b) (Fig. 4e). In contrast to AXOS, the maltodextrin supplementation period was associated with increased proportional abundance of the Bacteroidaceae family, and *Bacteroides* genus, in the *Prevotella*-plus group of volunteers only (LEfSe, *p* = 0.005). No genera or families were associated with maltodextrin supplementation in the *Prevotella*-minus group of volunteers using LEfSe (Additional file 4: Table S4). There was a weak, non-significant correlation between fibre intake and *Prevotella* proportional abundance in the *Prevotella*-plus group (R^2^ = 0.503, *p* = 0.074) (Fig. 5) but the mean fibre intake for the *Prevotella*-plus group was significantly higher (25.0 g/d) than for the *Prevotella*-minus group (19.1 g/d) (*p* = 0.025). Considerable inter-individual variation was observed between volunteers at the phylum and family level (Additional file 5: Fig. S1).

**Fig. 6:**
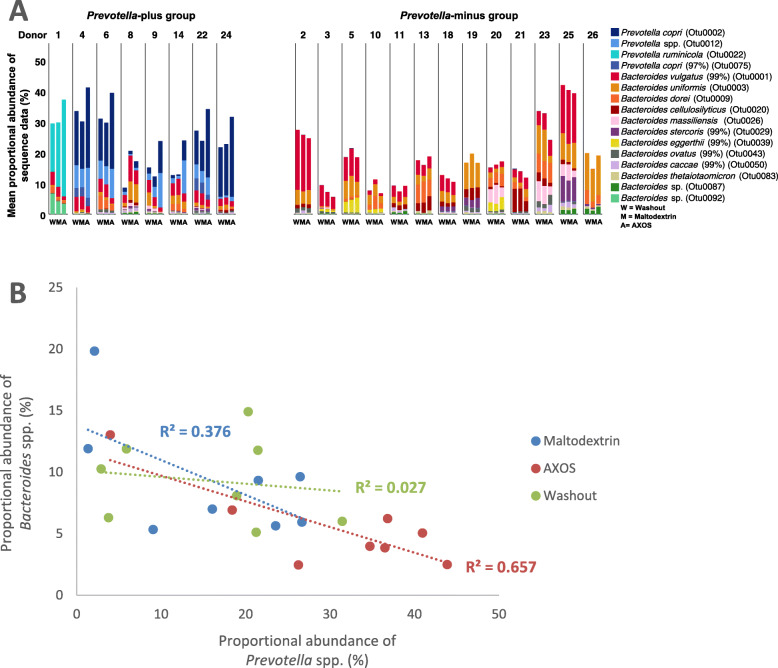
**a** Changes in the proportional abundance of selected *Bacteroides* spp. and *Prevotella* spp. operational taxonomic units (OTUs) per volunteer during the maltodextrin supplementation periods for the *Prevotella*-plus and *Prevotella*-minus groups and M = maltodextrin, A = AXOS supplementation periods, W = washout period. **b** Correlation between *Bacteroides* spp. and *Prevotella* spp. proportional abundance, for the *Prevotella*-plus group, during the initial washout period (R^2^ = 0.027, *p* = 0.698), AXOS supplement period (R^2^ = 0.657, *p* = 0.015) and maltodextrin supplementation period (R^2^ = 0.376, *p* = 0.106)

Seven out of the eight *Prevotella*-plus volunteers possessed a high proportional abundance of *Prevotella copri* (OTU0002, OTU0012 and OTU0075), which cumulatively accounted for up to around 40% of the sequence reads when certain volunteers were on the AXOS supplements, with the presence of multiple *Prevotella* OTUs in most of these volunteers. One of the volunteers (volunteer number 1) however had a high proportional abundance of *P. ruminicola* (OTU0022) rather than *P. copri.* (Fig. 6a). Of the top 100 out of 2563 OTUs defined using a cut off of 97% sequence similarity (each representing > 0.22% of the total number of sequences), the most abundant OTUs were identified as *P. copri* (OTU0002) and *Prevotella* spp. (OTU0012) in the *Prevotella-*plus group (Additional file 6: Table S5A). *Bacteroides vulgatus* (OTU0001) and *Bacteroides uniformis* (OTU0003) were most prevalent in the *Prevotella*-minus group of volunteers (Additional file 6: Table S5B). Overall, the proportional abundance of *Prevotella* spp*.* was negatively correlated with that of *Bacteroides* spp*.* across the eight *Prevotella*-plus group responders, but the anti-correlation only reached significance during the AXOS supplementation period (R^2^ = 0.657, *p* = 0.015) (Fig. 6b).

*Bifidobacterium longum* was detected in all baseline samples across the volunteers, with the exception of volunteer 24 (Fig. 4e and Additional file 6: Table S5) and *B. longum* (OTU0011) was found to be the most proportionally abundant species/OTU, followed by *Bifidobacterium adolescentis* (OTU0014). Moreover, when comparing the washout periods, two *Bifidobacterium* OTUs were more prevalent in the *Prevotella*-minus group, namely *B. longum* (OTU0011, *p* = 0.001) and *B. adolescentis* (OTU0014, *p* = 0.012). This may also help to explain the higher proportional abundance of *Anaerostipes hadrus* (OTU0015, *p* = 0.0001), a lactate utilising-butyrate producing species present in greater proportional abundance in the *Prevotella-*minus group (Additional file 3: Table S3), as bifidobacteria generate lactate as a fermentation end product.

### Bacterial community diversity indices

Bacterial community α-diversity was measured using the Shannon index. Across the entire cohort of volunteers there was no significant change in diversity during the AXOS supplementation period when compared to the maltodextrin and washout periods. Microbial community analysis of samples from the maltodextrin supplementation period showed similar diversity with the washout period (Additional file 7: Fig. S2). When looking at the data stratified by Prevotellaceae presence at baseline the Shannon diversity index was significantly lower during the AXOS supplementation period (compared with washout period) for the *Prevotella*-plus group of volunteers (Wilcoxon *p* < 0.04).

### Faecal short chain fatty acid levels

Faecal short chain fatty acids were measured for all volunteers (*n* = 21) at the beginning of the study (control), middle (intermediate supplement) and end (last supplement) of each of the two supplementation periods, and also at the end of the second supplement period after a washout period (washout visit 7), which gave a total of seven measurements per volunteer. There was considerable inter-individual variation in SCFA concentrations, with the total SCFA levels in the washout period ranging from 23.2 to 94.8 mM (Additional file 8: Fig. S3).

**Fig. 7:**
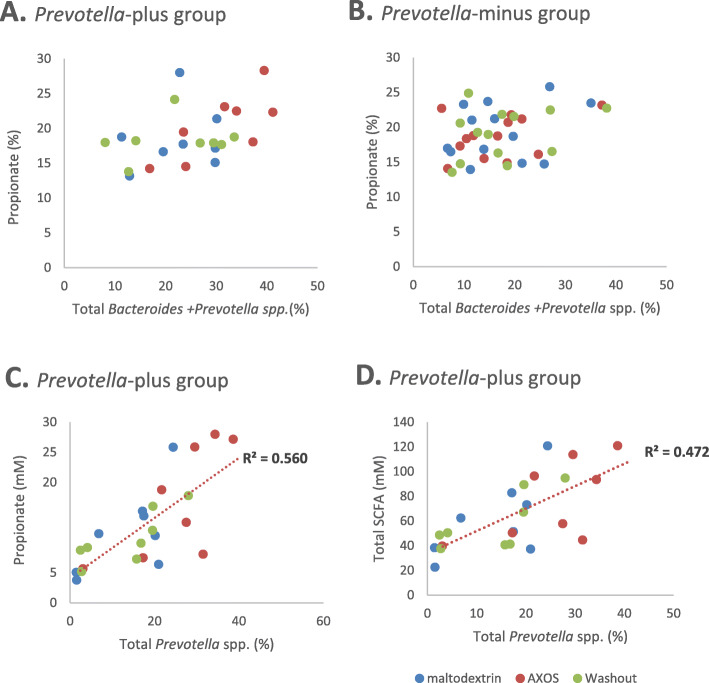
Correlation between the proportional abundance of *Bacteroides* and *Prevotella* spp. and propionate (%) during washout, maltodextrin and AXOS supplement periods for the (**a**) *Prevotella*-plus group and (**b**) *Prevotella*-minus group, (**c**) the correlation between proportional abundance of *Prevotella* spp. % and propionate concentration (mM) (*p* < 0.0001) and (**d**) the correlation between proportional abundance of *Prevotella* spp. % and total SCFA concentration (mM) (*p* < 0.0001)

Mean total SCFA concentrations for the 21 volunteers were increased by the AXOS supplement compared to the washout period (*p* = 0.002) and the maltodextrin supplement (*p* = 0.003). There was no significant change in proportional distribution of acetate, propionate or butyrate among total SCFA with either the dietary intervention, or with *Prevotella*-plus/minus status (Additional file 9: Fig. S4) There was no significant association between propionate percentage of total SCFA and the proportional abundance of *Prevotella* and *Bacteroides* spp. (Fig. 7a and b). However, within the *Prevotella*-plus group, the relative abundance of *Prevotella* spp. showed a significant positive correlation with both propionate (*p* < 0.0001) and total SCFA (*p* < 0.0001) concentrations (Fig. 7c and d respectively).

### Faecal calprotectin levels

Faecal calprotectin levels were measured in the faecal samples from the washout, maltodextrin and AXOS supplementation periods and there were no differences between the three periods across all the volunteers (*p* = 0.448). There were no significant differences in calprotectin levels between the AXOS and maltodextrin periods, or between the *Prevotella*-plus/minus groups of volunteers. (Additional file 10: Fig. S5).

### Serum cholesterol, glucose, HDL, LDL, triglyceride levels and blood pressure measurements

Blood serum parameters, glucose, HDL, LDL and triglycerides, were measured at the beginning and end of each supplementation period (*n* = 20, as one volunteer did not provide blood samples). There were no significant differences between the four periods (first supplement washout, second supplement washout, maltodextrin and AXOS supplements) in either the levels of cholesterol, glucose, HDL cholesterol, LDL cholesterol, triglyceride and the LDL-HDL ratios for the *Prevotella*-plus and *Prevotella*-minus groups (Additional file 11: Fig. S6).

## Discussion

This study investigated the impact of wheat bran AXOS on faecal microbiota and short chain fatty acid profiles in a human supplementation study. Here, the composition of the microbial communities was influenced by the nature of the dietary supplements. Maltodextrin resulted in relatively modest changes in gut microbial composition in comparison with AXOS. Furthermore, it did not result in an increase in faecal calprotectin levels although other studies, mostly conducted in rodents, have reported that maltodextrin supplements may promote gut inflammation [29]. During the AXOS supplementation period the changes occurred rapidly, within the first sampling point (at day 5), as has been reported in other studies [1, 2, 15, 16, 30]. *Bifidobacterium* species were significantly stimulated in the AXOS supplement period, in agreement with previous studies [27, 31, 32]. *B. longum* was identified as the predominant species within the bifidobacteria and was significantly more proportionally abundant during the AXOS supplementation period compared to baseline (*p* = 0.022) and maltodextrin supplementation periods (*p* = 0.015) for all volunteers. This may be important as bifidobacteria are generally considered beneficial to health and their proportional abundance is often diminished in certain disease states and in the elderly, particularly the frail elderly [33]. As such, AXOS may be considered a viable prebiotic option in elderly cohorts, where the aim is to promote the growth of bifidobacteria. It is important to note, however, that bifidobacteria are not the only bacterial group to be promoted by this substrate and conclusive evidence on their benefits for health is still needed.

Crucially, in this study we show that the microbiota responses to AXOS supplementation depended on the initial composition of the microbiota (*Prevotella*-plus or *Prevotella*-minus groupings). There was large inter-individual variation in the composition of the gut microbiota in the baseline samples with eight out of the 21 volunteers exhibiting a *Prevotella* dominant microbiota whilst the other volunteers showed a *Bacteroides* dominant population. An anti-correlation between *Prevotella* and *Bacteroides* spp. has been reported previously, with some authors referring to these as ‘enterotypes’ [12, 34–36]. Since it is not yet established that these gut microbial communities conform to a common microbiota composition we prefer to avoid applying the term ‘enterotype’ and this view is strongly supported by an extensive recent analysis of metagenome data [37].

The increase in *Prevotella* proportional abundance during the AXOS supplementation period suggests a possible dietary cause for the *Prevotella*-plus and -minus microbiota profiles [36]. Previously, it was suggested that *Bacteroides* spp. predominance was associated with animal fat and protein based diets and *Prevotella* spp. were associated with diets containing higher amounts of dietary fibre [11, 12, 38]. Our results suggest that AXOS may be one fibre type that favours the growth of *Prevotella* within the human gut.

It has also been shown that *Prevotella-*predominant faecal microbiota profiles tend to be associated with rapid gut transit as assessed by Bristol stool scores [39]. In the current study habitual dietary fibre intake was slightly higher overall, but not significantly, in the *Prevotella-*plus than in the *Prevotella-*minus individuals and the percentages of *Prevotella* correlated with total SCFA concentrations in faeces for the washout samples. Faecal SCFA concentrations have been shown to increase with the rate of gut transit in humans, and gut transit is promoted by fibre intake [39]. On the other hand, the habitual fibre intakes of *Prevotella*-minus and *Prevotella*-plus individuals showed considerable overlap in our study and there was no significant correlation overall between fibre intake and the relative abundance of *Prevotella*. This might reflect individual differences in gut transit that are independent of fibre intake, or other unknown host factors. Alternatively, it may be that there is some form of mutual antagonism between the major *Bacteroides* and *Prevotella* species within the human gut microbiota that tends to preclude their coexistence. *Prevotella* spp. have also been found to predominate over *Bacteroides* spp. in the microbiota of rural communities including hunter gatherers [11, 40].

Overall, these data suggest that *Prevotella* outcompeted *Bacteroides* for the supplementary AXOS in the *Prevotella*-plus group of volunteers, since *Bacteroides* proportional abundance decreased significantly with AXOS. Interestingly, bifidobacteria were significantly stimulated by AXOS in both *Prevotella*-plus and *Prevotella*-minus volunteers. This suggests that both *Prevotella* and certain bifidobacteria (notably *B. longum)* were able to benefit from the presence of AXOS. On the other hand, bifidobacteria were initially less proportionally abundant in *Prevotella*-plus than in the *Prevotella*-minus volunteers, and their AXOS-stimulated proportional abundances correspondingly lower.

In this study the response to AXOS supplements was dependent on the initial composition of the volunteers’ microbiota when stratified into *Prevotella*-plus and -minus groups. The health consequences of increasing the dominance of *Prevotella* spp. are still largely unknown [41]. Evidence is mixed and may be context dependent. For example, *P. copri* has been reported to be associated with several inflammatory conditions including the onset of rheumatoid arthritis [42] and ankylosing spondylitis [43]. In contrast, a microbiota that is dominated by *Prevotella* spp. is associated with increased weight loss on long term diets [44] and, separately, Gu et al. [45] reported that diabetic patients dominated by *Prevotella* exhibited greater improvements in metabolic parameters following acarbose treatment compared to those dominated by *Bacteroides.* Furthermore, in children in low to middle income countries, *Prevotella* species are anti-correlated with diarrhoeal disease, indicating that they may have a protective role [38].

Given the limited number of cultured isolates and reference genomes available for *P. copri* there is relatively little genotypic and phenotypic information available on this bacterium [46]. Recent whole shotgun metagenomics-based work has, however, indicated that *P. copri* is more diverse than considered previously, and is comprised of four distinct clades [47]. This inherent diversity may be an important driver of the opposing health impacts associated with *P. copri* and is worthy of further investigation. Interestingly, in the current study there were individuals that harboured multiple distinct *Prevotella* OTUs, indicating that these clades may not be mutually exclusive. However, given the comparatively lower resolution of partial 16S rRNA gene sequences, whole shotgun metagenomics-based studies, and attempts to culture and genome sequence key isolates, could provide more detailed information regarding whether specific sub-types of *Prevotella* species might respond to AXOS-based dietary interventions, and their potential subsequent impacts on health.

We also found that the *Prevotella*-plus and *Prevotella*- minus groups differ in the proportional abundance of many other bacterial OTUs that have potential health consequences. In particular, bifidobacteria were less proportionally abundant both before and after AXOS-stimulation in the *Prevotella*-plus group. Taken together, these conflicting associations suggest that improved prebiotic interventions may need to be stratified in future, to ensure optimal promotion of target organisms, and to avoid promoting the growth of bacterial groups that may be associated with ill-health. In the future, microbiota profiling is therefore likely to become an important tool that will be needed for personalised nutritional [48] and medical advice [42, 45].

## Conclusions

This study revealed rapid changes in faecal microbiota composition in response to consuming wheat bran AXOS supplements. In particular, *Bifidobacterium* species which are commonly associated with health, were significantly stimulated by AXOS, as reported previously [49–51]. Importantly, the results also revealed considerable inter-individual variation in the composition of the volunteer’s faecal microbiota. Approximately 40% of the volunteers supported a *Prevotella* dominant microbiota compared to a *Bacteroides* dominant profile, and in these individuals the AXOS supplements favoured proliferation of the *Prevotella* genus, which correlated with a significant increase in total faecal SCFA levels. Given the inter-individual variation in responses, microbiota profiling should therefore be considered when testing the efficacy of both existing and novel prebiotics in the future.

## Methods

### Volunteer recruitment and medical health screening

A study brochure, radio advertisement and notice boards were used to promote the study. Inclusion criteria for the study were as follows: both male and female volunteers were recruited to the study if they were aged 60 years and above, with a body mass index (BMI) 20–32 kg/m^2^ which is within the normal to slightly obese BMI range. The volunteers were invited for medical screening with a GP using medical health screening questionnaires. Exclusion criteria were as follows: Intolerance to fructose or any of the ingredients in the prebiotic mix, on prescription antibiotics within the past 3 months, bowel disorders, vegetarian or vegan, eating disorders and food intolerances, wheat and gluten allergy, coeliac disease, alcohol and/or other substance abuse, regular intake of prebiotic or probiotic supplements, smoking, psychiatric disorders resulting in perceived inability to give informed consent, lipid/cholesterol lowering medication (Additional file 1: Table S1A).

### Human dietary supplements

Participants consumed their habitual diet throughout the controlled, cross-over, randomised, double-blinded study. Test products were packaged in similar wrappings. The AXOS and maltodextrin supplements were taken orally in 5 g amounts, three times daily with meals. The study was approved by the Ethics Committee of the Rowett Institute, University of Aberdeen (study number HSMC/15/004). This trial was registered at clinicaltrials.gov no. NCT02693782.

Eligible volunteers (metadata provided in Additional file 1: Table S1) were randomised to receive either AXOS or maltodextrin as their first supplement. The wheat bran extract provided by Cargill Inc. was comprised of arabinoxylan oligosaccharides (AXOS; 71%) with an average degree of polymerisation (DP) of 5 and the β-glucan content was 15%. The maltodextrin (C Dry MD 01910) contained 95% carbohydrate, of which 4% were sugars with a dextrose equivalent (DE) of 12–16. Following the initial 5 days of maintenance diet (their habitual diet), the volunteers provided faecal samples and blood samples for analysis at regular intervals (Fig. 1). The study ended with a 5-day washout period.

### Food frequency (FFQ) and gastrointestinal tolerance questionnaires

Volunteers were requested to complete semi-quantitative and validated [52] Food Frequency Questionnaires (FFQ) at the start and end of the study (Scottish Collaborative Group version 6.6, University of Aberdeen, Aberdeen, UK) (Fig. 1). Food consumption was recorded as measures per day and then number of days per week. Nutrient content of the calculated daily consumption of the food were derived by entering the McCance & Widdowsons’ food code and weight per day into nutrient analysis package Windiets [53].

### Gastrointestinal tolerance

Gastrointestinal tolerance was determined using a daily questionnaire, with volunteers requested to record any gastrointestinal changes. Bowel movements were recorded as number of movements between 0 and 3 or greater than 3. Stool appearance was recorded as normal, hard or watery.

### Faecal sample collection, preparation and storage

Volunteers were provided with faecal sample collection kits that contained a leak proof bag. The samples were processed within 5 h of collection by manually mixing the sample for 1–2 min to give an even consistency. Aliquots (5 g) of the mixed faecal samples were placed into Dispomix tubes (MACS Miltenyl Biotec), with 5 ml of PBS buffer containing 30% glycerol. The Dispomix tubes were then homogenised by mixing at 3900 rpm, for 5 s forward and reverse over 65 s. The homogenised samples were aliquoted into lysing matrix E tubes in 0.45 ml volumes prior to DNA extraction using the FastDNA Spin kit for Soil (MP Biomedicals, Illkirch, France). For SCFA analysis, 1.5 ml sterile water was added to 0.5 g of each sample and then vortex mixed for 2 to 3 mins until the sample was homogenised. These were then centrifuged at 1500 g for 10 mins to pellet debris, and 1 ml of supernatant was removed and stored at -20 °C for subsequent SCFA analysis.

### DNA extractions from human faecal samples

DNA was extracted immediately from Dispomix prepared samples using the FastDNA Spin kit (MP Biomedicals, Illkirch, France). For each sample collected, 450 μl was placed in lysing matrix E tubes and 978 μl of sodium phosphate buffer and 122 μl MT buffer were added to each tube, which was processed following manufacturer’s instructions. The DNA was eluted in 50 μL FastPrep elution buffer.

### PCR amplification and Illumina MiSeq sequencing

The extracted DNA was used as a template for PCR amplification of the V1-V2 region of bacterial 16S rRNA genes using the barcoded fusion primers MiSeq-27F (5′-AATGATACGGCGACCACCGAGATCTACACTATGGTAATTCCAGMGTTYGATYMTGGCTCAG-3′) and MiSeq-338R (5′-CAAGCAGAAGACGGCATACGAGAT-barcode-AGTCAGTCAGAAGCTGCCTCCCGTAGGAGT-3′, which also contain adaptors for down-stream Illumina MiSeq sequencing as described previously [9]. Each sample was amplified with a unique (12 base) barcoded reverse primer.

PCR amplification was undertaken with Q5 High-fidelity DNA Polymerase (New England BioLabs). Each reaction mix contained DNA template (1 μl), 5 x Q5 Buffer (5 μl), 10 mM dNTPs (0.5 μl), 10 μM F primer (1.25 μl), 10 μM R primer (1.25 μl), Q5 Taq (0.25 μl) and sterile, deionised water (15.75 μl) to a final volume of 20 μl. The PCR amplification conditions were as follows: 2 min at 98 °C; 20 cycles of 30 s at 98 °C, 30 s at 50 °C, 120 s at 72 °C; ending with 5 mins at 72 °C then a holding temperature at 10 °C. Four PCR reactions were prepared for each DNA sample as described previously [9]. Following confirmation of adequate and appropriately sized product by gel electrophoresis, the quadruplicate reactions were pooled and ethanol precipitated and then quantified using a Qubit 2.0 Fluorometer (Life Technologies Ltd). A sequencing master-mix was created using equimolar concentrations of DNA from each sample. Sequencing was carried out on an Illumina MiSeq machine, using 2 × 250 bp read length, at the Wellcome Sanger Institute (Cambridgeshire, UK). Sequence data has been deposited in the European Nucleotide Archive and is available under study accession number ERP015431, and sample accession numbers ERS1138028 to ERS1138179 (Additional file 2: Table S2).

The sequences obtained were analysed using the Mothur software package [54] with the forward and reverse reads assembled into paired read contigs. Any paired contigs that were shorter than 270 bp, longer than 480 bp, contained ambiguous bases or contained homopolymeric stretches of longer than 7 bases were then removed. Unique sequences were grouped together and aligned against the SILVA reference database. Pre-clustering (diffs = 3) was performed to reduce the impact of sequencing errors. Rare sequences were removed (cut off = 5) and the OTUs were generated at a 97% similarity cut-off level using the default OptiClust setting in mothur. Reads from chloroplast, mitochondria, archaea, eukaryote and unknown sequences were removed from the dataset [55]. As a result, the final dataset had a total of 1,290,544 sequences with a range of 3928–40,532 sequences per sample. All samples were rarefied to 3928 reads to ensure equal sequencing depth for all comparisons (average Good's coverage at this sequence depth was 98%). The final OTU-level results are shown in Additional file 6: Table S5 A, B. Metastats analysis [56] was used to determine any OTUs that were significantly different between two sample cohorts (washout versus AXOS), and *p*-values were corrected as described previously [57] to allow for the false discovery rate over multiple comparisons. Significant associations across all cohorts were identified using LEfSe analysis [58]. The Shannon diversity index was used to calculate bacterial diversity per sample.

### Short chain fatty acid (SCFA) analysis

SCFA levels in faecal samples were measured by gas chromatography as described previously [59]. Following derivatisation of the samples using N- tert-butyldimethylsilyl-N-methyltrifluoroacetamide, the samples were analysed using a Hewlett Packard gas chromatograph fitted with a fused silica capillary column with helium as the carrier gas.

### Faecal calprotectin analysis

Faecal calprotectin levels were measured using The CALPRO Calprotectin ELISA test (ALP) (CALPRO AS, Norway) following the manufacturer’s instructions. Briefly, approximately 100 mg of faeces was mixed with 4.9 ml diluted faecal extraction buffer. Diluted sample extracts and controls were dispensed into the plate wells coated in immunoaffinity-purified polyclonal rabbit antibodies specific for calprotectin and incubated at room temperature on a horizontal plate shaker for 45 mins at 500–700 rpm. The wells were washed and 100 μl enzyme substrate solution added per well, then 100 μl 1 M NaOH was added to stop the reaction. The plates were read at an optical density of 405 nm using an ELISA plate reader and values used to calculate calprotectin concentrations.

### Blood glucose, cholesterol, HDL, LDL, triglyceride measurement

Whole blood was collected in EDTA vacutainers. A small aliquot was set aside at room temperature for lipid profile and glucose measurements immediately. Total cholesterol, high density lipids (HDL) cholesterol, low density lipids (LDL) cholesterol, non-HDL cholesterol, triglyceride and glucose were measured using Alere Cholestech LDX® LIPIS PROFILE GLU cassettes and analysed using an Alere Cholestech LDX® reader, following the manufacturer’s instructions.

### Statistical analysis

We aimed for a minimum of twenty subjects since previous studies have demonstrated that this is a sufficient sample size for significance for short chain fatty acids and microbiota analysis [15], and twenty-one volunteers completed this study. Sequencing and SCFA data from these experiments were analysed by ANOVA with donor, time and substrate within donor as random effects, and with substrate, time and their interaction as fixed effects. When an effect was significant (*P* < 0.05) mean values were then compared by post-hoc t-test based on the output from the ANOVA analysis. The Benjamini-Hochberg test [57] was applied to correct for false discovery rate. For validation purposes, the Wilcoxon signed-rank test was applied to data that showed significance using LEfSe and Metastats analysis.

## Supplementary information

**Additional file 1 Table S1**. Inclusion and exclusion criteria for the study and individual volunteer metadata .

**Additional file 2 Table S2**. Proportional abundance of each OTU (in %) per sample (97% OTU cut-off)

**Additional file 3 Table S3**. Metastats analysis of the most proportionally abundant operational taxonomic units OTUs (i.e. those accounting for > 0.5% of total proportional abundance) between individuals within the *Prevotella*-plus group washout period and those in the *Prevotella*- minus group during the washout period.

**Additional file 4 Table S4**. LEfSe analysis of samples from the AXOS, maltodextrin and washout periods, at the genus and family level for (A) *Prevotella*-plus and (B) *Prevotella*-minus groups

**Additional file 5 Figure S1**. Average changes in bacterial proportional abundance per individual volunteer at the (A) phylum and (B) family level

**Additional file 6 Table S5**. Metastats analysis of the most proportionally abundant operational taxonomic unit OTUs (i.e. those accounting for > 0.5% of total proportional abundance) between washout and AXOS supplement periods for (A) *Prevotella*-plus and (B) *Prevotella*-minus

**Additional file 7 Figure S2**. Mean bacterial diversity, as assessed using the Shannon diversity index, across all volunteers for each dietary supplementary period for both the *Prevotella*-plus and *Prevotella*-minus groups

**Additional file 8 Figure S3**. Concentration (mM) of short chain fatty acids during the washout, maltodextrin and AXOS periods for both *Prevotella*-plus and *Prevotella*-minus groups, for (A, B) total SCFA, (C, D) acetate, (E, F) propionate and (G, H) butyrate.

**Additional file 9 Figure S4**. Mean acetate, propionate and butyrate concentrations (mM) measured from faecal samples in the combined washout, AXOS supplementation and maltodextrin supplementation periods for the (A) *Prevotella*-plus group, (B) *Prevotella-* minus group and percentage SCFA for the (C) *Prevotella-*plus group, and (D) *Prevotella-*minus group.

**Additional file 10 Figure S5**. Calprotectin levels for all collected volunteer faecal samples

**Additional file 11 Figure S6**. Mean blood sample measurements across volunteers for the malotdextrin and AXOS periods with the corresponding washout periods, measuring cholesterol, glucose, HDL cholesterol, LDL cholesterol, non-HDL cholesterol, triglyceride, total cholesterol/HDL ratio and LDL/HDL ratio, for the (A) *Prevotella* plus group and (B) *Prevotella* minus group

## Data Availability

Volunteer metadata provided in Additional file 1: Table S1 and faecal microbiota sequence data has been deposited in the European Nucleotide Archive and is available under study accession number ERP015431, and sample accession numbers ERS1138028 to ERS1138179 (Additional file 2: Table S2).
