# Supplementary material for: Relative abundance of the Prevotella genus within the human gut microbiota of elderly volunteers determines the inter-individual responses to dietary supplementation with wheat bran arabinoxylan-oligosaccharides
Source: BMC Microbiol. 2020 Sep 14;20:283. doi: 10.1186/s12866-020-01968-4 (PMC7490872; doi:10.1186/s12866-020-01968-4)
Supplement: Supplementary file 1 — Additional file 1 Table S1. Inclusion and exclusion criteria for the study and individual volunteer metadata . [file 12866_2020_1968_MOESM1_ESM.pdf]

**Additional file 1: Table S1A.** List of inclusion and exclusion criteria for volunteers screening.

***Inclusion Criteria***

- Males and females aged 60 years and above
- Body mass index 20-32 kg/$m^2$

***Exclusion Criteria***

- Fructose intolerance/ or any of the ingredients in the prebiotic mix
- On prescription antibiotics within the past 3 months
- Bowel disorder
- Vegetarian or vegan
- Eating disorders and food intolerances (restricted eating)
- Wheat and gluten allergy, coeliac disease
- Alcohol and/or other substance abuse
- Regular intake of prebiotic or probiotic supplements
- Smoking
- Psychiatric disorders resulting in perceived inability to give informed consent (including severe depression, lithium treatment, schizophrenia, severe behavioural disorders)
- Lipid/Cholesterol lowering medication (as cholesterol was one of the endpoints of the study)

**Additional file 1: Table S1B.** Individual volunteer metadata of height, weight, BMI, gender, supplement group and number of faecal samples provided in the study out of a total of seven, for volunteers ranging from 60 to 74 years old. Supplement group 1 intervention direction, Placebo to AXOS. Supplement group 2 intervention direction, AXOS to Placebo. Out of the twenty-one volunteers, eight are in the *Prevotella*-plus group and thirteen are in the *Prevotella*-minus group. *All volunteers provided 7 out of 7 faecal samples with the exception of volunteer 008 who provided 5.

| Volunteer | Height (m) | Weight (kg) | BMI (kg/m2) | Gender | Supplement Group | *Prevotella* group |
|---|---|---|---|---|---|---|
| **001** | 1.63 | 65.05 | 24.47 | M | 1 | Plus |
| **002** | 1.62 | 82.25 | 31.25 | F | 2 | Minus |
| **003** | 1.69 | 66.80 | 23.18 | F | 1 | Minus |
| **004** | 1.75 | 81.00 | 26.33 | M | 2 | Plus |
| **005** | 1.65 | 77.10 | 28.05 | M | 2 | Minus |
| **006** | 1.63 | 58.10 | 21.71 | F | 2 | Plus |
| **008*** | 1.49 | 49.25 | 21.94 | F | 1 | Plus |
| **009** | 1.61 | 57.70 | 22.18 | M | 1 | Plus |
| **010** | 1.58 | 64.95 | 25.78 | F | 2 | Minus |
| **011** | 1.59 | 58.40 | 23.08 | F | 1 | Minus |
| **013** | 1.66 | 68.85 | 24.77 | F | 2 | Minus |
| **014** | 1.59 | 58.65 | 23.09 | F | 2 | Plus |
| **018** | 1.71 | 88.20 | 30.18 | M | 2 | Minus |
| **019** | 1.77 | 71.60 | 22.67 | M | 2 | Minus |
| **020** | 1.63 | 50.85 | 18.98 | F | 2 | Minus |
| **021** | 1.63 | 59.05 | 22.23 | F | 1 | Minus |
| **022** | 1.74 | 87.25 | 28.77 | M | 1 | Plus |
| **023** | 1.62 | 78.00 | 29.51 | F | 1 | Minus |
| **024** | 1.57 | 67.10 | 27.18 | F | 2 | Plus |
| **025** | 1.74 | 79.90 | 26.43 | M | 2 | Minus |
| **026** | 1.59 | 69.20 | 27.10 | F | 1 | Minus |
