# Supplementary material for: Relative abundance of the Prevotella genus within the human gut microbiota of elderly volunteers determines the inter-individual responses to dietary supplementation with wheat bran arabinoxylan-oligosaccharides
Source: BMC Microbiol. 2020 Sep 14;20:283. doi: 10.1186/s12866-020-01968-4 (PMC7490872; doi:10.1186/s12866-020-01968-4)
Supplement: Supplementary file 3 — Additional file 3 Table S3. Metastats analysis of the most proportionally abundant operational taxonomic units OTUs (i.e. those accounting for > 0.5% of total proportional abundance) between individuals within the Prevotella-plus group washout period and those in the Prevotella- minus group during the washout period. [file 12866_2020_1968_MOESM3_ESM.pdf]

Additional file 3: Table S3 Metastats analysis of the most proportionally abundant operational taxonomic units (OTUs; those accounting for > 0.5 % of total proportional abundance between *Prevotella*- plus group individual washout period and *Prevotella*- minus group individual washout period). P-values less than p<0.001 are shown in bold. P values were corrected using the Benjamini-Hochberg method (Benjamini, Hochberg 1995) to account for multiple comparisons.

OTU	MegaBLAST Closest Match (Representative Seq.)	No.of seq	Mean of Prevotella-plus group washout (%)	Mean of Prevotella-minus group washout (%)	p-value	Significant after applying Benjamini-Hochberg correction
Otu0001	*Bacteroides vulgatus* (99%)	27146	3.80	5.66	0.075	No
Otu0002	*Prevotella copri*	21446	8.15	0.03	**0.001**	Yes
Otu0003	*Bacteroides uniformis*	20348	1.59	4.97	**0.001**	Yes
Otu0004	*Faecalibacterium prausnitzii* (99%)	18279	3.08	3.59	0.365	No
Otu0005	*Faecalibacterium prausnitzii*	10941	1.87	1.86	0.972	No
Otu0006	*Ruminococcus bicirculans* (99%)	9984	1.63	1.79	0.778	No
Otu0007	*Guyana massiliensis*	9460	0.54	2.01	0.011	Yes
Otu0008	*Faecalibacterium prausnitzii* (98%)	8876	1.16	1.75	0.086	No
Otu0009	*Bacteroides dorei*	8472	1.16	1.90	0.146	No
Otu0010	*Faecalibacterium prausnitzii* (99%)	8456	1.30	1.33	0.939	No
Otu0011	*Bifidobacterium longum*	8396	0.36	1.55	**0.001**	Yes
Otu0012	*Prevotella* spp.	8221	2.99	0.01	**0.001**	Yes
Otu0013	*Escherichia coli*	7427	3.64	0.07	0.143	No
Otu0014	*Bifidobacterium adolescentis*	7359	0.44	1.16	0.012	Yes
Otu0015	*Anaerostipes hadrus*	6716	0.58	1.47	**0.001**	Yes
Otu0016	*Barnesiella intestinihominis*	6647	1.58	0.92	0.088	No
Otu0017	*Faecalibacterium prausnitzii*	6603	1.03	1.21	0.415	No
Otu0018	Uncharacterised *Ruminococcaceae*	6600	1.12	1.18	0.922	No
Otu0019	*Streptococcus salivarius*	6261	0.27	1.65	0.002	Yes
Otu0020	*Bacteroides cellulosilyticus*	6160	0.14	1.69	**0.001**	Yes
Otu0021	*Eubacterium rectale*	6048	0.69	1.09	0.120	No
Otu0022	*Prevotella ruminicola*	5974	2.05	0.00	0.043	No
Otu0023	*Subdoligranulum* sp. (97%)	4820	0.42	1.19	0.006	Yes
Otu0024	Uncharacterised *Rikenellaceae*	4808	0.01	1.18	0.054	No
Otu0025	*Collinsella aerofaciens*	4242	0.71	0.73	0.941	No
Otu0026	*Bacteroides massiliensis*	4218	0.23	1.14	0.033	No
Otu0027	*Sutterella wadsworthensis*	4097	0.46	0.95	0.189	No
Otu0028	*Alistipes putredinis*	4079	0.83	0.72	0.308	No
Otu0029	*Bacteroides stercoris*(99%)	3941	0.24	1.02	0.051	No
Otu0030	*Ruminococcus bromii* (99%)	3844	0.88	0.70	0.544	No
Otu0031	*Bifidobacterium faecale/adolescentis*	3722	0.27	0.53	0.117	No
Otu0032	*Romboutsia timonensis* (99%)	3437	0.74	0.57	0.335	No
Otu0033	Uncharacterised *Alphaproteobacteria*	3385	0.57	0.91	0.782	No
Otu0034	*Collinsella aerofaciens*	3261	0.28	0.72	0.041	No
Otu0035	*Roseburia faecis*	3103	0.57	0.43	0.263	No
Otu0036	*Blautia* sp. (99%)	3091	0.22	0.66	**0.001**	Yes
Otu0037	*Intestinibacter bartlettii* (98%)	3010	1.06	0.35	**0.001**	Yes
Otu0038	Uncharacterised *Firmicutes*	2960	0.00	0.87	**0.001**	Yes
Otu0039	*Bacteroides eggerthii* (99%)	2927	0.05	0.63	0.006	Yes
Otu0040	Uncharacterised *Rikenellaceae*	2912	0.00	0.74	0.062	No
Otu0041	*Bifidobacterium pseudocatenulatum*	2874	0.06	0.51	0.056	No
Otu0042	*Bifidobacterium catenulatum*(98%)	2866	0.10	0.47	0.060	No
Otu0043	*Bacteroides ovatus* (99%)	2809	0.30	0.59	0.028	No
Otu0044	Uncharacterised *Ruminococcaceae*	2807	0.90	0.27	0.005	Yes
Otu0045	*Faecalibacterium prausnitzii* (97%)	2738	0.56	0.43	0.403	No
Otu0046	*Blautia* sp. (99%)	2624	0.22	0.47	0.003	Yes
Otu0047	*Fusicatenibacter saccharivorans* (97%)	2502	0.20	0.53	0.001	Yes

Otu0048	*Subdoligranulum* sp.	2377	0.59	0.35	0.129	No
Otu0049	*Alistipes onderdonkii*	2372	0.11	0.65	0.003	Yes
Otu0051	*Catenibacterium mitsuokai* (99%)	2286	0.91	0.00	**0.001**	Yes
Otu0052	*Sutterella massiliensis*	2221	0.90	0.10	0.008	Yes
Otu0053	Uncharacterised *Rikenellaceae*	2217	0.00	0.58	0.081	No
Otu0054	*Sutterella* sp.	2132	0.00	0.55	**0.001**	Yes
Otu0058	Uncharaterised *Bacteria*	2079	0.90	0.02	**0.001**	Yes
Otu0059	*Parabacteroides merdae*	2075	0.11	0.58	0.002	Yes
Otu0060	*Faecalibacterium prausnitzii* (98%)	2059	0.58	0.24	0.059	No
Otu0062	*Eubacterium siraeum* (99%)	1962	0.06	0.53	0.070	No
Otu0063	Uncharacterised *Clostridiales*	1959	0.07	0.51	0.066	No
Otu0067	Uncharacterised *Erysipelotrichaceae*	1914	0.72	0.19	0.199	No
Otu0072	*Dialister invisus*	1821	0.61	0.05	0.041	No
Otu0073	*Phascolarctobacterium* sp. (99%)	1783	0.61	0.22	0.090	No
Otu0075	*Prevotella copri* (97%)	1669	0.89	0.01	0.003	Yes
Otu0079	*Alistipes* spp.	1565	0.55	0.16	0.166	No
Otu0092	*Bacteroides* spp.	1344	0.85	0.00	**0.001**	Yes
Otu0100	Uncharacterised *Bacteria*	1238	0.49	0.01	0.174	No
Otu0106	Uncharacterised *Bacteroidetes*	1204	0.66	0.00	0.001	Yes
