# Supplementary material for: Relative abundance of the Prevotella genus within the human gut microbiota of elderly volunteers determines the inter-individual responses to dietary supplementation with wheat bran arabinoxylan-oligosaccharides
Source: BMC Microbiol. 2020 Sep 14;20:283. doi: 10.1186/s12866-020-01968-4 (PMC7490872; doi:10.1186/s12866-020-01968-4)
Supplement: Supplementary file 4 — Additional file 4 Table S4. LEfSe analysis of samples from the AXOS, maltodextrin and washout periods, at the genus and family level for (A) Prevotella-plus and (B) Prevotella-minus groups [file 12866_2020_1968_MOESM4_ESM.pdf]

Additional file 4 Table S4A. LEfSe analysis at the genus and family levels, between the different sample cohorts – AXOS, maltodextrin and washout period in the *Prevotella*-plus group, only include taxa >0.5 % of total proportional abundance. Cohorts in the supplement column indicate where taxa were associated with a particular supplement. Bacterial groups showing significant differences during the AXOS supplementation period were validated using the Wilcoxon test

Genus level	pValue	Supplement
Prevotella	0.002	AXOS
Bacteroides	0.005	maltodextrin
Faecalibacterium		-
Ruminococcaceae_unclassified		-
Ruminococcus		-
Clostridiales_unclassified		-
Escherichia/Shigella		-
Oscillibacter	0.013	maltodextrin
Bifidobacterium	<0.0001	AXOS
Alistipes		-
Subdoligranulum		-
Roseburia		-
Firmicutes_unclassified		-
Blautia		-
Dialister		-
Bacteria_unclassified		-
Barnesiella		-
Lachnospiraceae_unclassified		-
Catenibacterium		-
Sutterella		-
Collinsella		-
Intestinibacter		-
Clostridium_sensu_stricto		-
Turicibacter		-
Dorea		-
Anaerostipes		-
Holdemanella		-
Coprococcus		-
Fusicatenibacter		-
Phascolarctobacterium		-
Romboutsia		-
Bacteroidetes_unclassified		-
Ruminococcus2	0.042	maltodextrin
Proteobacteria_unclassified	0.031	Washout
Ehallii		-
Parabacteroides		-
Guyana		-

Family level	pValue	Supplement
Ruminococcaceae		-
Prevotellaceae	0.002	AXOS
Lachnospiraceae		-
Bacteroidaceae	0.005	maltodextrin
Enterobacteriaceae		-
Erysipelotrichaceae		-
Clostridiales_unclassified		-
Porphyromonadaceae		-
Bifidobacteriaceae	<0.0001	AXOS
Rikenellaceae		-
Firmicutes_unclassified		-
Peptostreptococcaceae		-
Veillonellaceae		-
Sutterellaceae		-
Coriobacteriaceae		-
Bacteria_unclassified		-
Clostridiaceae_1		-
Acidaminococcaceae		-
Bacteroidetes_unclassified		-
Proteobacteria_unclassified	0.033	Washout

Wilcoxon signed-ranked test	Genus						Family					
	Prevotella			*Bifidobacterium*			*Prevotellaceae*			*Bifidobacteriaceae*		
	Washout	AXOS	diff	Washout	AXOS	diff	Washout	AXOS	diff	Washout	AXOS	diff
1	28.03	31.21	3.18	0.56	2.70	2.14	29.71	33.32	3.62	0.56	2.70	2.14
4	30.93	43.25	12.32	1.91	5.45	3.54	32.03	44.14	12.12	1.91	5.45	3.54
6	12.02	33.58	21.56	2.21	14.05	11.84	12.50	33.71	21.21	2.21	14.05	11.84
8	2.37	3.05	0.69	2.98	1.53	-1.45	2.37	3.05	0.69	2.98	1.53	-1.45
9	8.10	21.54	13.44	2.04	4.35	2.32	8.81	22.00	13.19	2.04	4.35	2.32
14	2.72	7.43	4.71	0.00	7.89	7.89	2.72	7.43	4.71	0.00	7.89	7.89
22	17.41	36.20	18.79	0.59	7.92	7.33	17.41	36.20	18.79	0.59	7.92	7.33
24	13.42	33.53	20.11	1.48	4.10	2.62	13.54	33.58	20.04	1.48	4.10	2.62
Median			12.04			4.78			11.97			4.78
Wilcoxon Stats			36			35			36			35
P-value			0.014			0.021			0.014			0.021

Additional file 4 Table S4B. LEfSe analysis at the genus and family levels, between the different sample cohorts – AXOS, maltodextrin and washout period in the *Prevotella*-minus group, only include taxa >0.5 % of total proportional abundance. Cohorts in the supplement column indicate where taxa were associated with a particular supplement. Bacterial groups showing significant differences during the AXOS supplementation period were validated using the Wilcoxon test

**Genus Level**	**pValue**	**Supplement**
Bacteroides		-
Faecalibacterium		-
Bifidobacterium	<0.0001	AXOS
Ruminococcaceae_unclassified		-
Subdoligranulum		-
Alistipes		-
Clostridiales_unclassified		-
Ruminococcus		-
Blautia		-
Roseburia		-
Guyana		-
Firmicutes_unclassified		-
Oscillibacter		-
Rikenella		-
Streptococcus		-
Lachnospiraceae_unclassified		-
Anaerostipes		-
Collinsella		-
Parabacteroides	0.031	Washout
Fusicatenibacter		-
Parasutterella		-
Sutterella		-
Ehallii		-
Barnesiella		-
Proteobacteria_unclassified		-
Dorea		-
Clostridium_IV		-
Alphaproteobacteria_unclassified		-
Ruminococcus2		-
Erysipelotrichaceae_unclassified		-
Bacteroidetes_unclassified		-
Bacteroidales_unclassified		-
Clostridium_XlVa		-
Phascolarctobacterium		-

**Family level**	**pValue**	**Supplement**
Ruminococcaceae		-
Bacteroidaceae		-
Lachnospiraceae		-
Bifidobacteriaceae	<0.0001	AXOS
Rikenellaceae		-
Clostridiales_unclassified		-
Erysipelotrichaceae		-
Porphyromonadaceae		-
Sutterellaceae		-
Coriobacteriaceae		-
Firmicutes_unclassified		-
Streptococcaceae		-
Peptostreptococcaceae		-
Proteobacteria_unclassified		-
Alphaproteobacteria_unclassified		-
Acidaminococcaceae		-
Bacteroidetes_unclassified		-
Bacteroidales_unclassified		-
Prevotellaceae		-

Wilcoxon signed-ranked test	Genus *Bifidobacterium* spp.			Family Bifidobacteriaceae		
	Washout	AXOS	diff	Washout	AXOS	diff
2	3.46	21.64	18.18	3.46	21.64	18.18
3	6.01	25.99	19.98	6.01	25.99	19.98
5	1.83	13.39	11.56	1.83	13.39	11.56
10	1.43	18.05	16.62	1.43	18.05	16.62
11	0.79	13.95	13.16	0.79	13.95	13.16
13	0.03	0.28	0.25	0.03	0.28	0.25
18	8.45	9.37	0.92	8.45	9.37	0.92
19	7.41	11.69	4.28	7.41	11.69	4.28
20	11.20	22.66	11.46	11.20	22.66	11.46
21	3.13	15.02	11.89	3.13	15.02	11.89
23	3.64	16.93	13.29	3.64	16.93	13.29
25	4.91	13.26	8.35	4.91	13.26	8.35
26	6.77	16.88	10.11	6.77	16.88	10.11
Median			11.23			11.23
Wilcoxon Stats			91			91
P-value			0.002			0.002
