# Supplementary material for: Relative abundance of the Prevotella genus within the human gut microbiota of elderly volunteers determines the inter-individual responses to dietary supplementation with wheat bran arabinoxylan-oligosaccharides
Source: BMC Microbiol. 2020 Sep 14;20:283. doi: 10.1186/s12866-020-01968-4 (PMC7490872; doi:10.1186/s12866-020-01968-4)
Supplement: Supplementary file 6 — Additional file 6 Table S5. Metastats analysis of the most proportionally abundant operational taxonomic unit OTUs (i.e. those accounting for > 0.5% of total proportional abundance) between washout and AXOS supplement periods for (A) Prevotella-plus and (B) Prevotella-minus [file 12866_2020_1968_MOESM6_ESM.pdf]

Additional file 6: Table S5A Metastats analysis of the most proportionally abundant operational taxonomic units (OTUs accounting for > 0.5 % of total proportional abundance between washout and AXOS supplement periods) for the *Prevotella*-plus volunteer group. P-values less than p<0.001 are shown in bold. P values were corrected using the Benjamini-Hochberg method (Benjamini, Hochberg 1995) to account for multiple comparisons.

OTU	MegaBLAST Closest Match (Representative Seq.)	No. of seq	Mean of Washout (%)	Mean of AXOS (%)	p-value	Significant after applying Benjamini-Hochberg correction
Otu0002	*Prevotella copri*	21373	8.15	15.64	0.036	No
Otu0012	*Prevotella* spp.	8193	2.99	6.49	0.022	No
Otu0001	*Bacteroides vulgatus* (99%)	7311	3.80	2.01	**0.001**	Yes
Otu0013	*Escherichia coli*	7060	3.64	2.76	0.678	No
Otu0004	*Faecalibacterium prausnitzii* (99%)	6238	3.08	2.84	0.645	No
Otu0022	*Prevotella ruminicola*	5971	2.05	4.20	0.464	No
Otu0005	*Faecalibacterium prausnitzii*	3748	1.87	1.46	0.315	No
Otu0016	*Barnesiella intestinihominis*	3475	1.58	1.51	0.868	No
Otu0006	*Ruminococcus bicirculans* (99%)	3258	1.63	1.21	0.448	No
Otu0003	*Bacteroides uniformis*	3110	1.59	0.98	0.404	No
Otu0010	*Faecalibacterium prausnitzii* (99%)	2841	1.30	1.60	0.502	No
Otu0008	*Faecalibacterium prausnitzii* (98%)	2525	1.16	1.23	0.825	No
Otu0051	*Catenibacterium mitsuokai* (99%)	2273	0.91	0.97	0.911	No
Otu0018	Uncharacterised *Ruminococcaceae*	2271	1.12	1.10	0.970	No
Otu0017	*Faecalibacterium prausnitzii*	2223	1.03	1.08	0.832	No
Otu0058	Uncharacterised *Bacteria*	1995	0.90	0.51	0.309	No
Otu0037	*Intestinibacter bartlettii* (98%)	1978	1.06	0.64	0.140	No
Otu0009	*Bacteroides dorei*	1889	1.16	0.57	0.144	No
Otu0052	*Sutterella massiliensis*	1808	0.90	0.63	0.478	No
Otu0030	*Ruminococcus bromii* (99%)	1698	0.88	0.53	0.290	No
Otu0028	*Alistipes putredinis*	1670	0.83	0.64	0.104	No
Otu0044	Uncharacterised *Ruminococcaceae*	1663	0.90	0.50	0.068	No
Otu0075	*Prevotella copri* (97%)	1660	0.89	0.58	0.485	No
Otu0072	*Dialister invisus*	1621	0.61	1.04	0.392	No
Otu0032	*Romboutsia timonensis* (99%)	1601	0.74	0.67	0.694	No
Otu0014	*Bifidobacterium adolescentis*	1555	0.44	1.58	0.037	No
Otu0021	*Eubacterium rectale*	1532	0.69	0.76	0.798	No
Otu0035	*Roseburia faecis*	1470	0.57	0.97	0.144	No
Otu0025	*Collinsella aerofaciens*	1437	0.71	0.62	0.665	No
Otu0015	*Anaerostipes hadrus*	1407	0.58	0.77	0.496	No
Otu0092	*Bacteroides* spp.	1344	0.85	0.44	0.484	No
Otu0067	Uncharacterised *Erysipelotrichaceae*	1329	0.72	0.41	0.502	No
Otu0100	Uncharacterised *Bacteria*	1223	0.49	0.89	0.568	No
Otu0106	Uncharacterised *Bacteroidetes*	1203	0.66	0.46	0.566	No
Otu0045	*Faecalibacterium prausnitzii* (97%)	1176	0.56	0.55	0.941	No
Otu0060	*Faecalibacterium prausnitzii* (98%)	1170	0.58	0.58	0.988	No
Otu0011	*Bifidobacterium longum*	1151	0.36	0.93	0.007	No
Otu0073	*Phascolarctobacterium* sp. (99%)	1126	0.61	0.44	0.592	No
Otu0007	*Guyana massiliensis*	1118	0.54	0.31	0.067	No
Otu0079	*Alistipes* spp.	1098	0.55	0.41	0.695	No
Otu0048	*Subdoligranulum* sp.	1096	0.59	0.48	0.602	No

Additional file 6: Table S5B Metastats analysis of the most proportionally abundant operational taxonomic units (OTUs accounting for > 0.5 % of total proportional abundance between washout and AXOS supplement periods) for the *Prevotella*-minus group. P-values less than p<0.001 are shown in bold. P values were corrected using the Benjamini-Hochberg method (Benjamini, Hochberg 1995) to account for multiple comparisons.

OTU	MegaBLAST Closest Match (Representative Seq.)	No. of seq	Mean of Washout (%)	Mean of AXOS (%)	p-value	Significant after applying Benjamini-Hochberg correction
Otu0001	*Bacteroides vulgatus* (99%)	19767	5.66	5.27	0.797	No
Otu0003	*Bacteroides uniformis*	16905	4.97	4.36	0.486	No
Otu0004	*Faecalibacterium prausnitzii* (99%)	12018	3.59	3.13	0.433	No
Otu0007	*Guyana massiliensis*	7764	2.01	1.61	0.584	No
Otu0005	*Faecalibacterium prausnitzii*	7179	1.86	2.25	0.289	No
Otu0011	*Bifidobacterium longum*	7174	1.55	3.14	**0.001**	Yes
Otu0006	*Ruminococcus bicirculans* (99%)	6719	1.79	2.01	0.714	No
Otu0009	*Bacteroides dorei*	6568	1.90	1.74	0.807	No
Otu0008	*Faecalibacterium prausnitzii* (98%)	6325	1.75	1.72	0.948	No
Otu0014	*Bifidobacterium adolescentis*	5787	1.16	3.13	0.003	No
Otu0020	*Bacteroides cellulosilyticus*	5757	1.69	1.37	0.488	No
Otu0019	*Streptococcus salivarius*	5715	1.65	1.61	0.953	No
Otu0010	*Faecalibacterium prausnitzii* (99%)	5579	1.33	1.90	0.230	No
Otu0015	*Anaerostipes hadrus*	5278	1.47	1.36	0.758	No
Otu0024	Uncharacterised *Rikenellaceae*	4799	1.18	1.56	0.756	No
Otu0021	*Eubacterium rectale*	4502	1.09	1.70	0.188	No
Otu0017	*Faecalibacterium prausnitzii*	4372	1.21	1.23	0.935	No
Otu0018	Uncharacterised *Ruminococcaceae*	4329	1.18	1.03	0.839	No
Otu0023	*Subdoligranulum* sp. (97%)	4055	1.19	0.99	0.485	No
Otu0026	*Bacteroides massiliensis*	3768	1.14	0.62	0.241	No
Otu0029	*Bacteroides stercoris* (99%)	3477	1.02	1.01	0.986	No
Otu0016	*Barnesiella intestinihominis*	3168	0.92	0.83	0.688	No
Otu0027	*Sutterella wadsworthensis*	3116	0.95	0.86	0.841	No
Otu0038	Uncharacterised *Firmicutes*	2952	0.87	0.93	0.852	No
Otu0031	*Bifidobacterium faecale/adolescentis*	2938	0.53	1.63	0.004	No
Otu0040	Uncharacterised *Rikenellaceae*	2912	0.74	0.91	0.887	No
Otu0033	Uncharacterised *Alphaproteobacteria*	2662	0.91	0.32	0.532	No
Otu0034	*Collinsella aerofaciens*	2634	0.72	0.69	0.896	No
Otu0025	*Collinsella aerofaciens*	2578	0.73	0.63	0.589	No
Otu0042	*Bifidobacterium catenulatum* (98%)	2552	0.47	1.45	0.183	No
Otu0036	*Blautia* sp. (99%)	2527	0.66	0.78	0.342	No
Otu0039	*Bacteroides eggerthii* (99%)	2514	0.63	0.82	0.610	No
Otu0041	*Bifidobacterium pseudocatenulatum*	2449	0.51	1.09	0.326	No
Otu0028	*Alistipes putredinis*	2406	0.72	0.60	0.266	No
Otu0053	Uncharacterised *Rikenellaceae*	2215	0.58	0.77	0.779	No
Otu0043	*Bacteroides ovatus* (99%)	2135	0.59	0.75	0.448	No
Otu0054	*Sutterella* sp.	2126	0.55	0.77	0.504	No
Otu0047	*Fusicatenibacter saccharivorans* (97%)	2122	0.53	0.69	0.380	No
Otu0030	*Ruminococcus bromii* (99%)	2121	0.70	0.37	0.088	No
Otu0049	*Alistipes onderdonkii*	2069	0.65	0.60	0.877	No
Otu0046	*Blautia* sp. (99%)	2020	0.47	0.66	0.205	No
Otu0063	Uncharacterised *Clostridiales*	1864	0.51	0.60	0.803	No
Otu0059	*Parabacteroides merdae*	1838	0.58	0.50	0.655	No
Otu0062	*Eubacterium siraeum* (99%)	1803	0.53	0.52	0.969	No
Otu0032	*Romboutsia timonensis* (99%)	1793	0.57	0.31	0.052	No
