# Supplementary material for: Relative abundance of the Prevotella genus within the human gut microbiota of elderly volunteers determines the inter-individual responses to dietary supplementation with wheat bran arabinoxylan-oligosaccharides
Source: BMC Microbiol. 2020 Sep 14;20:283. doi: 10.1186/s12866-020-01968-4 (PMC7490872; doi:10.1186/s12866-020-01968-4)
Supplement: Supplementary file 7 — Additional file 7 Figure S2. Mean bacterial diversity, as assessed using the Shannon diversity index, across all volunteers for each dietary supplementary period for both the Prevotella-plus and Prevotella-minus groups [file 12866_2020_1968_MOESM7_ESM.pdf]

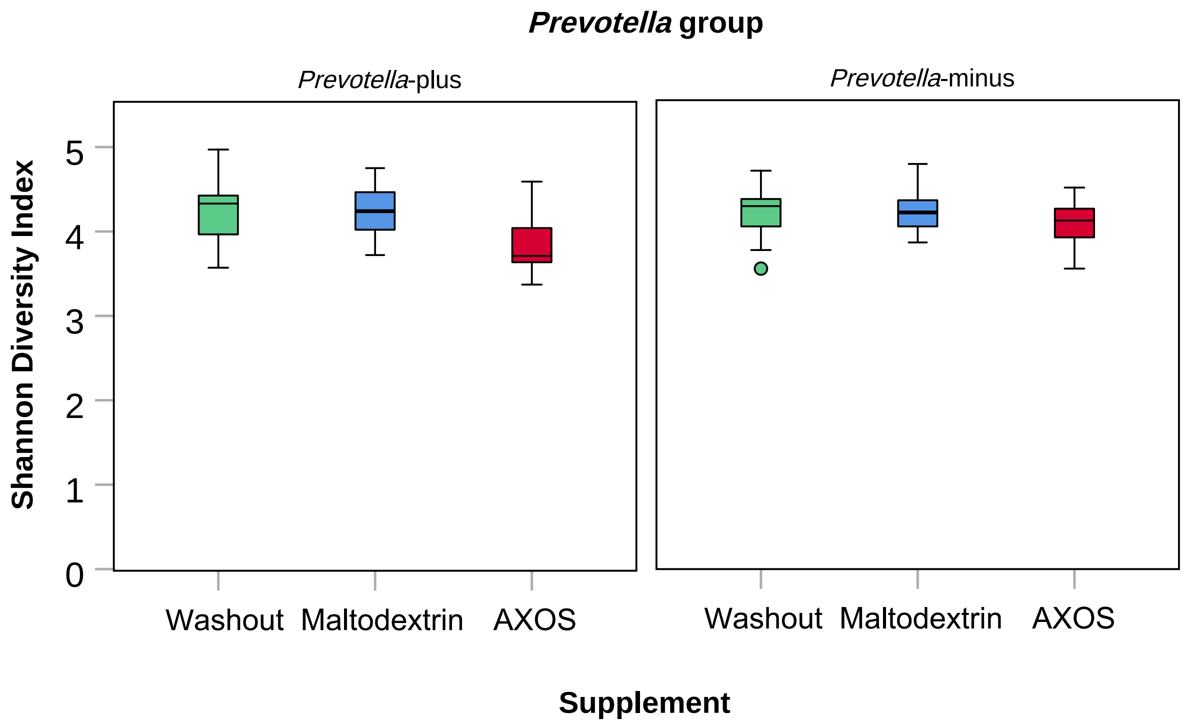

Additional file 7: Figure S2. Mean bacterial diversity across all volunteers for each dietary supplementary period for the *Prevotella*-plus and *Prevotella*-minus groups. There was significance different during the AXOS supplementation period (compared with washout period) for the *Prevotella*-plus group of volunteers (Wilcoxon $p<0.04$).
