# Supplementary material for: Relative abundance of the Prevotella genus within the human gut microbiota of elderly volunteers determines the inter-individual responses to dietary supplementation with wheat bran arabinoxylan-oligosaccharides
Source: BMC Microbiol. 2020 Sep 14;20:283. doi: 10.1186/s12866-020-01968-4 (PMC7490872; doi:10.1186/s12866-020-01968-4)
Supplement: Supplementary file 8 — Additional file 8 Figure S3. Concentration (mM) of short chain fatty acids during the washout, maltodextrin and AXOS periods for both Prevotella-plus and Prevotella-minus groups, for (A, B) total SCFA, (C, D) acetate, (E, F) propionate and (G, H) butyrate. [file 12866_2020_1968_MOESM8_ESM.pdf]

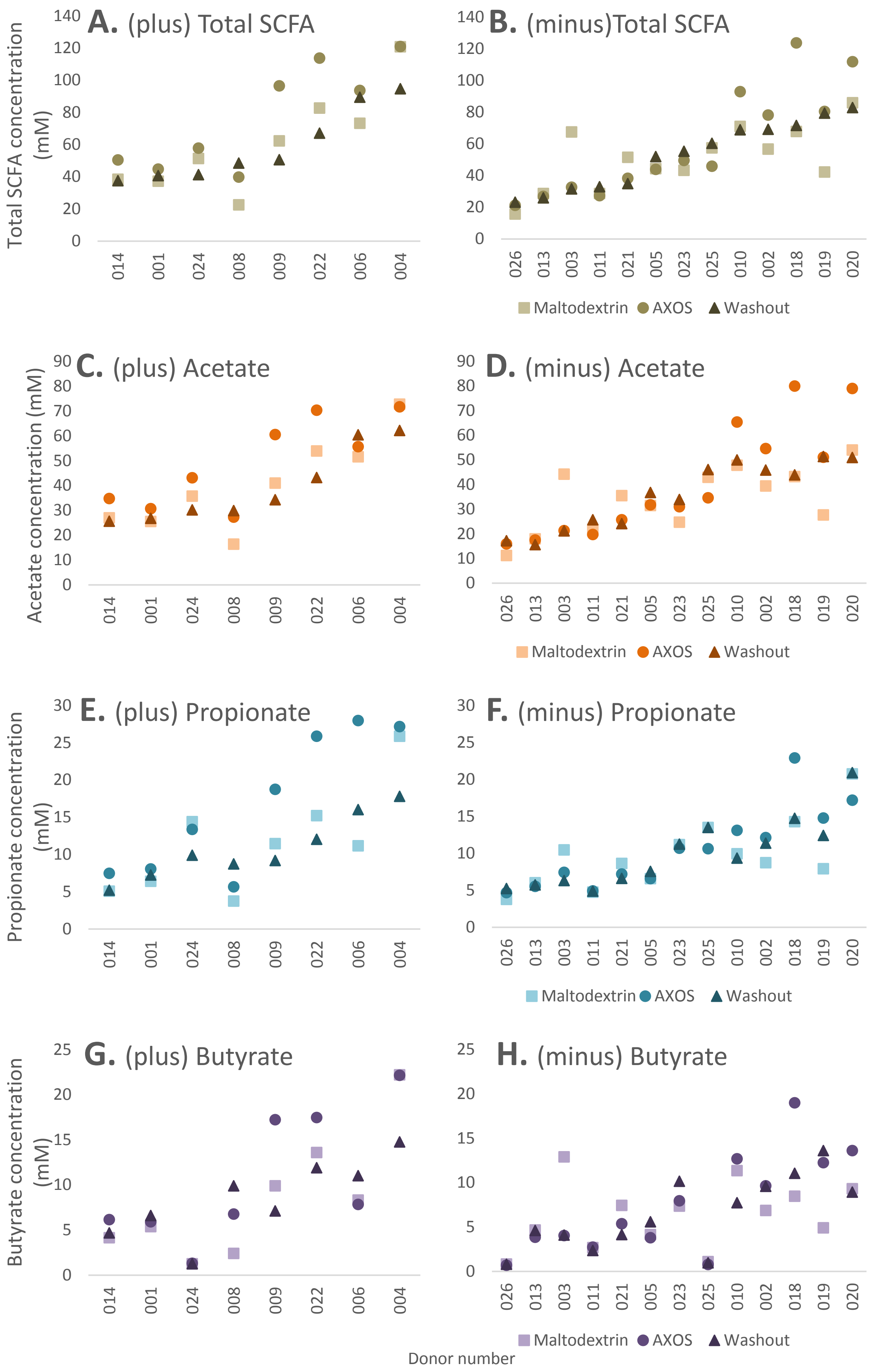

Additional file 8: Figure S3. Concentration of short chain fatty acids in faecal samples from the washout, maltodextrin and AXOS periods for both the *Prevotella-* plus and *Prevotella-* minus group, showing (A, B) total SCFA, (C, D) acetate, (E, F) propionate and (G, H) butyrate.
