# Supplementary material for: Relative abundance of the Prevotella genus within the human gut microbiota of elderly volunteers determines the inter-individual responses to dietary supplementation with wheat bran arabinoxylan-oligosaccharides
Source: BMC Microbiol. 2020 Sep 14;20:283. doi: 10.1186/s12866-020-01968-4 (PMC7490872; doi:10.1186/s12866-020-01968-4)
Supplement: Supplementary file 9 — Additional file 9 Figure S4. Mean acetate, propionate and butyrate concentrations (mM) measured from faecal samples in the combined washout, AXOS supplementation and maltodextrin supplementation periods for the (A) Prevotella-plus group, (B) Prevotella- minus group and percentage SCFA for the (C) Prevotella-plus group, and (D) Prevotella-minus group. [file 12866_2020_1968_MOESM9_ESM.pdf]

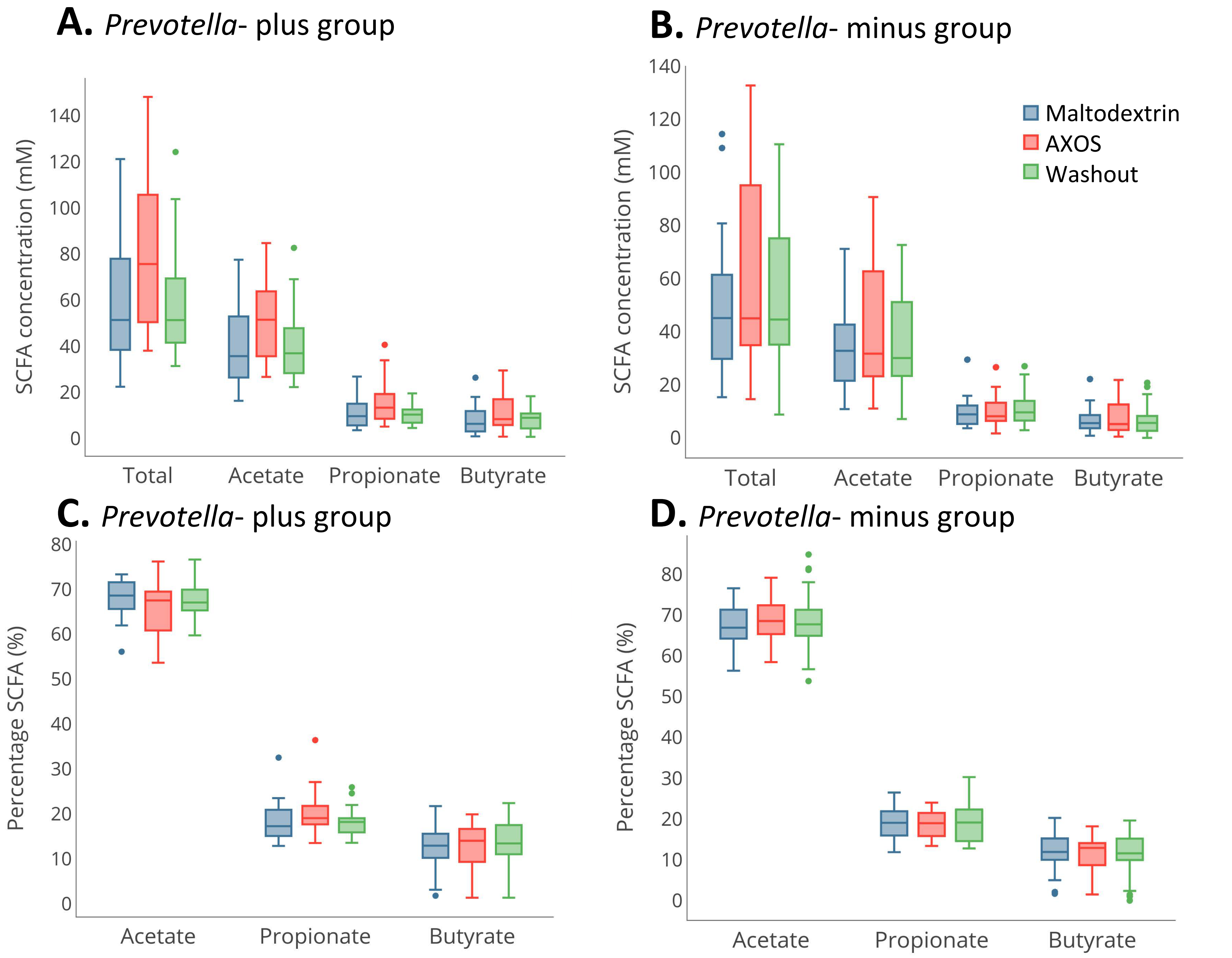

Add file 9: Fig S4. Mean acetate, propionate and butyrate concentrations (mM) measured from faecal samples in the combined washout, AXOS supplement and maltodextrin supplementation period for the (A) *Prevotella*-plus group, (B) *Prevotella*-minus group and percentage SCFA for the (C) *Prevotella*-plus group, and (D) *Prevotella*-minus group.
