# Supplementary material for: Relative abundance of the Prevotella genus within the human gut microbiota of elderly volunteers determines the inter-individual responses to dietary supplementation with wheat bran arabinoxylan-oligosaccharides
Source: BMC Microbiol. 2020 Sep 14;20:283. doi: 10.1186/s12866-020-01968-4 (PMC7490872; doi:10.1186/s12866-020-01968-4)
Supplement: Supplementary file 10 — Additional file 10 Figure S5. Calprotectin levels for all collected volunteer faecal samples [file 12866_2020_1968_MOESM10_ESM.pdf]

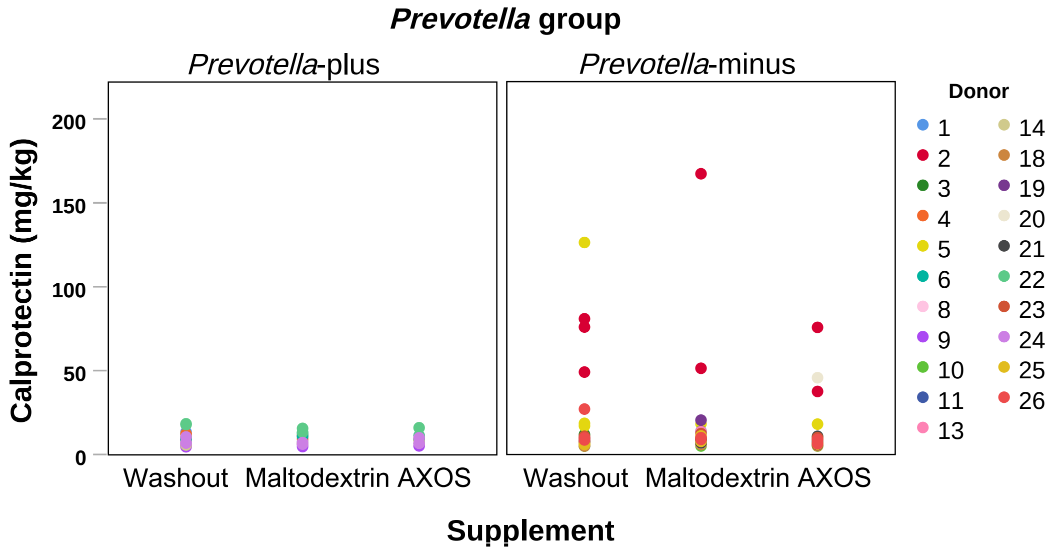

Additional file 10: Figure S5. Intestinal inflammation were measured by faecal calprotectin levels using faecal samples per visit per volunteer. No significant differences were found in calprotectin levels between the AXOS and maltodextrin period. Volunteer 002 showed positive values for calprotectin levels in all seven visits and volunteer 005 had one positive sample in the initial washout sample. Values within the normal range= 5-50 mg/kg (positive values with symptomatic inflammatory bowel disease would be expected to be in the range of 200-40,000 mg/kg.)
