# Supplementary material for: Relative abundance of the Prevotella genus within the human gut microbiota of elderly volunteers determines the inter-individual responses to dietary supplementation with wheat bran arabinoxylan-oligosaccharides
Source: BMC Microbiol. 2020 Sep 14;20:283. doi: 10.1186/s12866-020-01968-4 (PMC7490872; doi:10.1186/s12866-020-01968-4)
Supplement: Supplementary file 5 — Additional file 5 Figure S1. Average changes in bacterial proportional abundance per individual volunteer at the (A) phylum and (B) family level [file 12866_2020_1968_MOESM5_ESM.pdf]

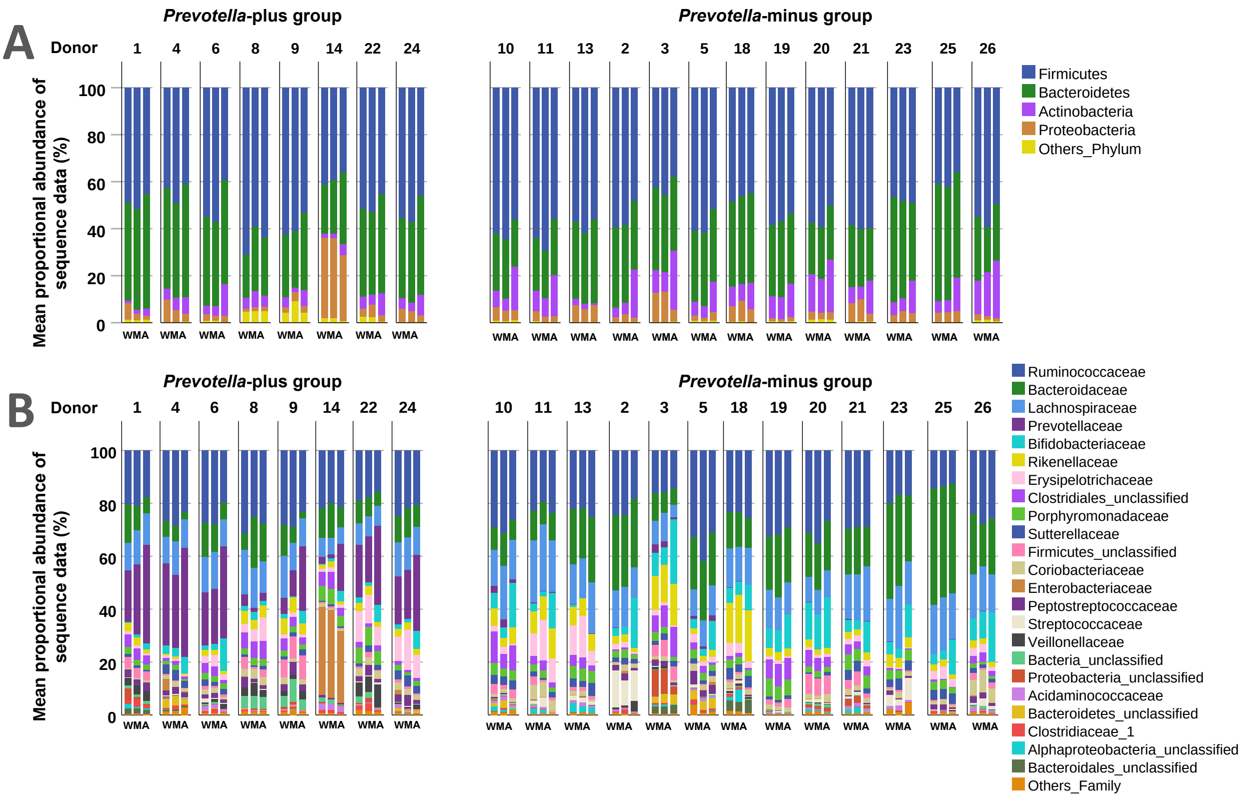

Additional file 5: Figure S1. Changes in average proportional abundance within individual volunteers at the bacterial (A) phylum and (B) family level based on Illumina MiSeq sequencing of 16S rRNA gene amplicons. W= washout period, M= Maltodextrin period, A= AXOS period.
