# Supplementary material for: Relative abundance of the Prevotella genus within the human gut microbiota of elderly volunteers determines the inter-individual responses to dietary supplementation with wheat bran arabinoxylan-oligosaccharides
Source: BMC Microbiol. 2020 Sep 14;20:283. doi: 10.1186/s12866-020-01968-4 (PMC7490872; doi:10.1186/s12866-020-01968-4)
Supplement: Supplementary file 11 — Additional file 11 Figure S6. Mean blood sample measurements across volunteers for the malotdextrin and AXOS periods with the corresponding washout periods, measuring cholesterol, glucose, HDL cholesterol, LDL cholesterol, non-HDL cholesterol, triglyceride, total cholesterol/HDL ratio and LDL/HDL ratio, for the (A) Prevotella plus group and (B) Prevotella minus group [file 12866_2020_1968_MOESM11_ESM.pdf]

A. *Prevotella*-plus group

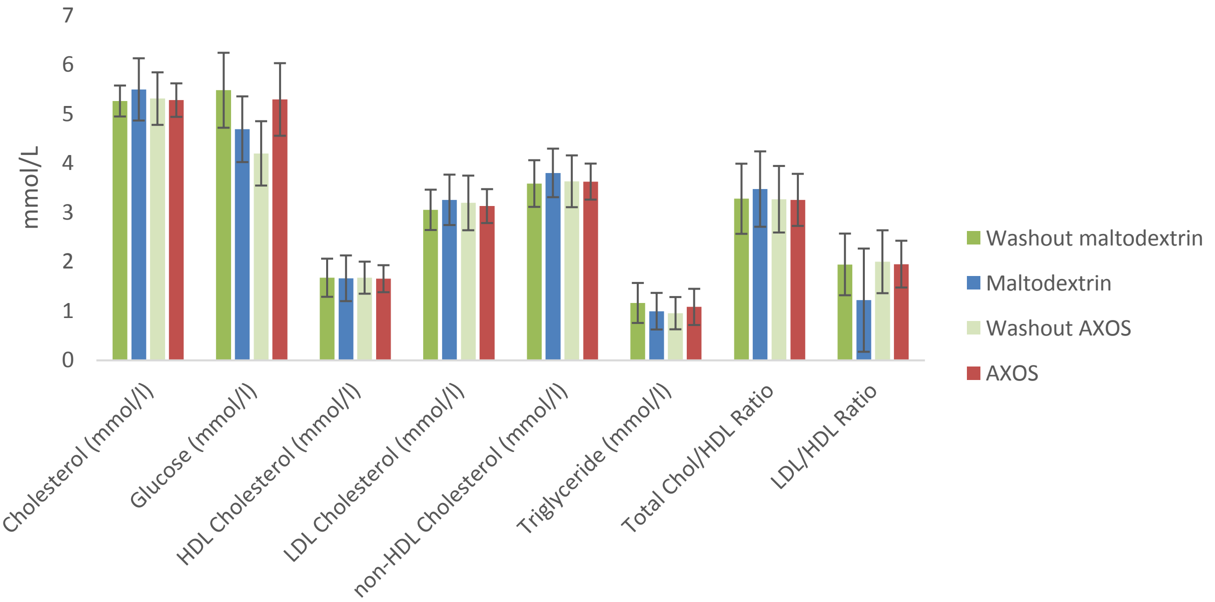

B. *Prevotella*- minus group

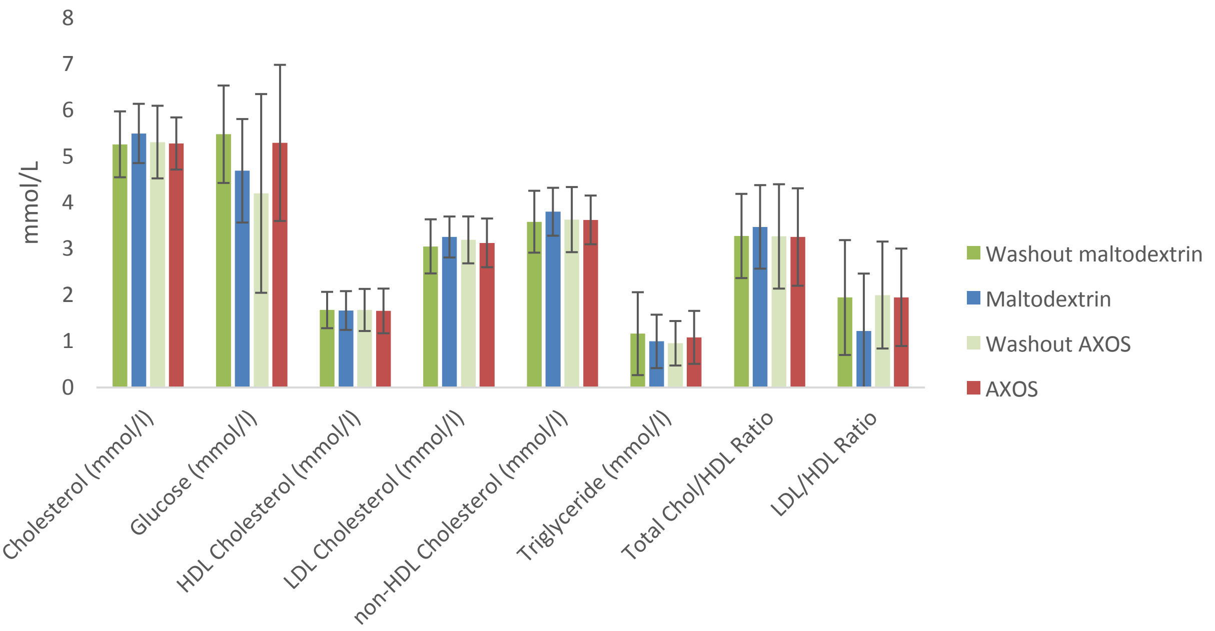

Additional file 11: Figure S6. Mean blood sample measurements across volunteers during the washout, maltodextrin and AXOS supplementation periods with the corresponding cholesterol, glucose, HDL cholesterol, LDL cholesterol, non-HDL cholesterol, triglyceride, total cholesterol/HDL ratio and LDL/HDL ratio, within the (A) *Prevotella*-plus and (B) *Prevotella*-minus groups.
